# Diet, but not food type, significantly affects micronutrient and toxic metal profiles in urine and/or plasma; a randomized, controlled intervention trial

**DOI:** 10.1093/ajcn/nqac233

**Published:** 2022-08-30

**Authors:** Leonidas Rempelos, Juan Wang, Marcin Barański, Anthony Watson, Nikolaos Volakakis, Catherine Hadall, Gultakin Hasanaliyeva, Eleni Chatzidimitriou, Amelia Magistrali, Hannah Davis, Vanessa Vigar, Dominika Średnicka-Tober, Steven Rushton, Kristin S Rosnes, Per O Iversen, Chris J Seal, Carlo Leifert

**Affiliations:** Lincoln Institute for Agri-Food Technology, University of Lincoln, Lincoln, United Kingdom; School of Agriculture, Food and Rural Development, Nafferton Ecological Farming Group, Newcastle University, Newcastle upon Tyne, United Kingdom; School of Agriculture, Food and Rural Development, Nafferton Ecological Farming Group, Newcastle University, Newcastle upon Tyne, United Kingdom; Human Nutrition Research Centre, Population and Health Sciences Institute, Newcastle University, Newcastle upon Tyne, United Kingdom; School of Agriculture and Biology, Shanghai Jiao Tong University, Shanghai, China; School of Agriculture, Food and Rural Development, Nafferton Ecological Farming Group, Newcastle University, Newcastle upon Tyne, United Kingdom; Laboratory of Neurobiology, Nencki Institute of Experimental Biology, Polish Academy of Sciences, Warsaw, Poland; Human Nutrition Research Centre, Population and Health Sciences Institute, Newcastle University, Newcastle upon Tyne, United Kingdom; Geokomi plc, Sivas-Faistos, Crete, Greece; Royal Victoria Infirmary, Newcastle upon Tyne Hospitals, Newcastle upon Tyne, United Kingdom; School of Agriculture, Food and Rural Development, Nafferton Ecological Farming Group, Newcastle University, Newcastle upon Tyne, United Kingdom; Department of Sustainable Crop and Food Protection, Faculty of Agriculture, Food and Environmental Sciences, Catholic University of the Sacred Heart, Piacenza, Italy; School of Agriculture, Food and Rural Development, Nafferton Ecological Farming Group, Newcastle University, Newcastle upon Tyne, United Kingdom; French Agency for Food, Environmental, and Occupational Health and Safety (ANSES), Maisons-Alfort, France; School of Agriculture, Food and Rural Development, Nafferton Ecological Farming Group, Newcastle University, Newcastle upon Tyne, United Kingdom; School of Agriculture, Food and Rural Development, Nafferton Ecological Farming Group, Newcastle University, Newcastle upon Tyne, United Kingdom; National Centre for Naturopathic Medicine, Southern Cross University, Lismore, New South Wales, Australia; Institute of Human Nutrition Sciences, Warsaw University of Life Sciences, Warsaw, Poland; Modelling Evidence and Policy Group, School of Natural and Environmental Sciences, Newcastle University, Newcastle upon Tyne, United Kingdom; Department of Nutrition, Institute of Medical Biology, University of Oslo, Oslo, Norway; Department of Nutrition, Institute of Medical Biology, University of Oslo, Oslo, Norway; Department of Haematology, Oslo University Hospital, Oslo, Norway; Human Nutrition Research Centre, Population and Health Sciences Institute, Newcastle University, Newcastle upon Tyne, United Kingdom; Department of Nutrition, Institute of Medical Biology, University of Oslo, Oslo, Norway; Plant Science, Southern Cross University, Lismore, New South Wales, Australia

**Keywords:** Western diets, Mediterranean diets, organic food, conventional food, phenolics, mineral micronutrients, cadmium, lead

## Abstract

**Background:**

Observational studies have linked Mediterranean Diets (MedDiets) and organic food consumption with positive health outcomes, which may be explained by higher mineral micronutrient and phenolic intake and lower dietary exposure to toxic compounds.

**Objectives:**

We aimed to determine the effects of diet and food type (organic compared with conventional) on urinary excretion (UE) and/or plasma concentrations of mineral micronutrients, phenolics, and toxic metals.

**Methods:**

Healthy adult participants were randomly allocated to a conventional (*n* = 14) or an intervention (*n* = 13) group. During a 2-wk period, the intervention group consumed a MedDiet made entirely from organic foods, whereas the conventional group consumed a MedDiet made from conventional foods. Before and after the intervention period, both groups consumed their habitual Western diets made from conventional foods. The primary outcome was UE and/or plasma concentrations of selected mineral micronutrients, toxic metals, and phenolic markers. In addition, we monitored diets using food diaries. The participants were aware of study group assignment, but the study assessors were not.

**Results:**

Changing from a Western Diet to a MedDiet for 2 wk resulted in significant increases in UE of total phenolics and salicylic acid (by 46% and 45%, respectively), the mineral micronutrients Co, I, and Mn (by 211%, 70%, and 102%, respectively), and the toxic metal Ni (by 42%), and plasma Se concentrations (by 14%). However, no significant effects of food type (organic compared with conventional) were detected. Redundancy analysis identified vegetables, coffee, wine, and fruit as positive drivers for UE of phenolic markers and mineral micronutrients, and fish consumption as a positive driver for UE of Cd and Pb.

**Conclusions:**

Although small effects of food type cannot be ruled out, our study suggests that only changing to a MedDiet with higher fruit and vegetable, and lower meat, consumption results in a large increase in phenolic and mineral micronutrient intakes.

This trial was registered at clinicaltrials.gov as NCT03254537.

## Introduction

The health benefits of increasing consumption of whole-grain products, fruits, and vegetables are well documented and are thought to be linked to increased intakes of fiber, phytochemicals (e.g., polyphenols and carotenoids), and minerals (e.g., Cu, Zn, and Se) with antioxidant and/or anti-inflammatory activity (1–3). In line with this, European consumers are advised to increase whole grain, fruit, and vegetable intakes, because consumption of all these food groups is currently below the level of WHO recommendations in most European Union (EU) countries (2, 4). This recommended dietary pattern is similar to the traditional Mediterranean diet (MedDiet), which has been referred to as the “gold standard in preventive medicine” because it is associated with a lower prevalence of mortality from all causes, and specifically lowers the incidence of certain types of cancer, cardiovascular diseases, type 2 diabetes, and neurodegenerative diseases (3, 5). Apart from increased whole grain, fruit, and vegetable consumption, the health benefits of the MedDiet have been linked with low intake of red meat, high fish and moderate wine consumption, and the use of olive oil (3).

More recently, observational studies have linked organic food consumption with lower prevalence of obesity, cancer, and a range of other diseases (6–12). However, there is uncertainty about these results due to the self-reported estimates of food consumption and the differences in lifestyles and diets between organic and conventional food consumers (13, 14). For example, organic consumers have higher intakes of whole grains, vegetables, and/or fruits, which may also explain the differences in the reported disease incidences (14–16). Studies into composition differences between organic and conventional crops reported slightly (15%–20%), but significantly, higher antioxidant capacity and (poly)phenol concentrations for a range of crops and higher mineral micronutrient (e.g., Cu, Se, Zn) but lower Cd concentrations in organic cereals (17–25). Higher concentrations of other nutritionally desirable phytochemicals (e.g., vitamin C, carotenoids) in organic crops than in conventional crops were also previously reported, but the differences were usually relatively small and/or less consistently detected (17–25). When organic and conventional meat and dairy products were compared, organic milk was found to have higher omega (ω)-3 fatty acid, iron, α-tocopherol, and carotenoid but lower iodine concentrations, and organic meat was reported to have higher ω-3 fatty acid concentrations (18, 26, 27). These composition differences may at least partially explain the positive health impacts of organic food consumption reported in observational studies (13, 14). However, it is important to note that both *1*) conventional compared with organic food consumption and *2*) diets with high fruit, vegetable, and whole-grain cereal intakes were also reported to significantly increase dietary pesticide exposure (14, 21, 28, 29).

The main objective of this randomized dietary intervention trial was to identify the relative effects of diet (habitual Western Diet compared with MedDiet) and food type (organic compared with conventional) on the urinary excretion (UE) and/or plasma concentrations of selected mineral micronutrients, toxic metals, salicylic acid, and total phenolics. Additional objectives were to assess diet composition and the strength of associations between diet components and UE of mineral micronutrients, toxic metals, and phenolic markers. Analyses focused on health-relevant compounds, for which systematic reviews/meta-analyses had reported relatively large differences between organic and conventional food crops, and in addition a wide range of mineral micronutrients and toxic metals which were not previously compared in organic and conventional crops (17, 18).

## Methods

### Study design

Full details of the study design have been reported previously (28). Briefly, the study was a 5-wk diet switch-over from a habitual Western Diet to a MedDiet, and then back to a habitual Western Diet with a nested, 2-wk, parallel-group, randomized, total diet replacement, dietary intervention trial comparing organic with conventional food consumption during the MedDiet phase.

The study (NCT03254537) was carried out in accordance with the Declaration of Helsinki. Ethical approval was obtained from the Faculty of Science, Agriculture and Engineering Research Ethics Committee, Newcastle University, United Kingdom (reference number: 17-SEA-017).

### Study participants

Twenty-seven healthy participants >18 y of age were recruited among postgraduate students participating in an agricultural field course in Crete (**Supplemental Figure 1**). They were enrolled by CL and CJS after the study design and aims were explained to them. The students were free to choose whether to take part and could leave the study without question at any stage. All participants gave written, informed consent before taking part in the study.

At baseline only, height, weight, and body fat percentage were measured using standard protocols (**Supplemental Table 1**) (28).

### Randomization and masking

Participants self-selected into small single-sex clusters based on shared accommodation during the intervention in Crete. Individuals within these clusters were then randomly allocated to the conventional (*n* = 14) or organic (*n* = 13) group by simple lottery. Clusters with 3 participants therefore had an uneven allocation, which resulted in a skewed sex balance between the study groups (Supplemental Table 1) (28).

Participants were aware of study group assignment because potential sensory differences between foods and color-coding of dishes of foods to ensure compliance made blinding impossible. All collected samples were coded until after biochemical and statistical analyses were completed. All data were analyzed according to group allocations without deviation from the protocol.

### Procedures

The whole study lasted 5 wk, with the 2-wk dietary intervention in Crete during weeks 2 and 3 (**Supplemental Figure 2**). During the pre- and postintervention periods, the participants stayed in Newcastle, United Kingdom and consumed their habitual, self-selected Western diets, which consisted entirely of conventional foods (except for 1 participant who consumed some organic milk).

During the intervention period, all participants consumed a defined MedDiet. The 7-d menu of foods and drinks provided at meals was the same for all participants, but the conventional group was provided with only conventional foods whereas the intervention group received only EU-certified organic foods. The menu was repeated in the second intervention week. Participants had free access to a selection of snacks and beverages including unrestricted amounts of bottled water in between meals [full details of the foods provided were previously published (28)].

The participants completed three 7-d food diary records (in weeks 1, 3, and 5) and we collected 3 fasting venous blood samples (15 mL, at the end of weeks 1, 3, and 5) and four 24-h urine samples (at the end of weeks 1, 2, 3, and 5) (Supplemental Figure 2) (28). **Table 1** describes the self-reported consumption of different foods/diet components with the habitual Western Diet in weeks 1 and 5 and the MedDiet in week 3 of the experiment.

**TABLE 1:** Self-reportedconsumption (portions/wk) of different foods/diet components by female and male participants consuming either habitual Western Diets or a defined MedDiet^1^

	Factors				ANOVA results (*P* values)		
	Sex		Diet		Main effects		
Food	Female (*n* = 39)	Male (*n* = 42)	MedDiet (*n* = 27)	Western (*n* = 54)	Sex	Diet	Interaction
Fruit and fruit juice	17.1 (14.9, 19.3)	15.9 (14.1, 17.8)	28.3 (26.0, 30.5)	10.6 (9.4, 11.8)	0.6940	<0.0001	0.5267
Vegetables (incl. potato)	21.1 (19.6, 22.6)	19.1 (17.8, 20.4)	25.4 (24.4, 26.5)	17.4 (16.1, 18.6)	0.3720	<0.0001	0.8725
Total fruit and vegetables	38.2 (35.2, 41.1)	35.0 (32.6, 37.5)	53.7 (51.4, 56.0)	28.0 (26.3, 29.7)	0.372	<0.0001	0.6983
Total refined cereals^2^	17.9 (16.2, 19.6)	23.5 (22.0, 25.1)	25.4 (23.6, 27.2)	18.5 (17.1, 20.0)	0.0777	0.0002	0.1954
Refined-flour bread	9.2 (7.9, 10.5)	16.3 (14.8, 17.8)	18.2 (16.4, 20.1)	10.2 (9.1, 11.4)	0.0098	<0.0001	0.1954
Total whole-grain cereals^2^	10.5 (9.0, 12.0)	7.4 (6.1, 8.8)	9.0 (7.5, 10.4)	8.9 (7.5, 10.2)	0.3170	0.9397	0.4516
Whole-grain bread	5.5 (4.3, 6.7)	5.0 (3.9, 6.0)	5.2 (4.0, 6.3)	5.2 (4.2, 6.3)	0.8354	0.9420	0.6562
HEPFs	6.8 (5.9, 7.7)	5.6 (5.0, 6.3)	7.1 (6.2, 8.0)	5.7 (5.1, 6.4)	0.4657	0.0908	0.8454
Meat	5.4 (4.7, 6.2)	7.6 (6.7, 8.5)	3.1 (2.8, 3.4)	8.3 (7.5, 9.1)	0.1311	<0.0001	0.0940
Fish	1.4 (1.2, 1.7)	1.0 (0.8, 1.2)	1.2 (1.0, 1.3)	1.2 (1.0, 1.4)	0.2900	0.8243	0.4608
Eggs	5.1 (4.4, 5.7)	6.9 (6.1, 7.6)	9.5 (8.8, 10.2)	4.2 (3.7, 4.8)	0.1257	<0.0001	0.2775
Cheese	5.1 (4.4, 5.8)	4.2 (3.7, 4.8)	7.2 (6.7, 7.8)	3.3 (2.8, 3.9)	0.4534	<0.0001	0.5530
Yogurt	2.3 (2.0, 2.7)	1.8 (1.6, 2.1)	2.3 (1.9, 2.7)	2.0 (1.7, 2.2)	0.3569	0.3911	0.3161
Total dairy products	13.0 (11.9, 14.2)	11.2 (10.2, 12.2)	14.8 (13.8, 15.9)	10.7 (9.8, 11.7)	0.3647	0.0018	0.4936
Total animal products	25.0 (23.5, 26.4)	26.6 (25.2, 28.0)	28.6 (27.3, 29.9)	24.4 (23.1, 25.7)	0.5179	0.0172	0.3805
Tea	3.8 (3.0, 4.7)	3.4 (2.8, 4.0)	0.6 (0.3, 1.0)	5.1 (4.4. 5.8)	0.7176	<0.0001	0.1185
Coffee	4.6 (3.9, 5.2)	4.6 (3.9, 5.2)	7.4 (6.5, 8.3)	3.2 (2.8, 3.6)	0.9969	<0.0001	0.3029
Beer	3.9 (3.1, 4.7)	4.8 (3.8, 5.8)	3.3 (2.7, 4.0)	4.9 (4.0, 5.8)	0.6108	0.1187	0.9100
Wine	6.8 (5.1, 8.7)	5.3 (3.7, 5.8)	15.2 (12.7, 17.6)	1.5 (0.9, 2.0)	0.4762	<0.0001	0.4121
Cider	0.7 (0.4, 1.0)	0.1 (0.0, 0.2)	0.0 (—)	0.6 (0.3, 0.8)	0.1687	0.0331	0.1123
Spirits	1.2 (0.8, 1.5)	1.0 (0.6, 1.3)	1.6 (0.9, 2.2)	0.8 (0.6, 1.0)	0.7436	0.1101	0.0769
Alcohol, units	12.6 (10.5, 14.7)	11.1 (9.0, 13.2)	20.1 (16.9, 23.3)	7.7 (6.5, 8.9)	0.7184	<0.0001	0.1826

1 Values are main effect means (95% CIs) unless indicated otherwise. HEPF, high-energy processed food; MedDiet, Mediterranean Diet.

2 Includes breakfast cereal products, bread, and all other cereal-based foods consumed.

Urine samples were assessed for 2 phenolic markers (salicylic acid and total phenolics), the toxic metals Al, Ba, Be, Cd, Ni, Pb, and Sb, the nontoxic metal Ti, and the mineral micronutrients Co, Cr, Cu, I, Mn, Mo, Se, and Zn. Plasma samples were assessed for Cu, Fe, Se, and Zn concentrations. Concentrations of Ba, Be, and Sb were below the limit of detection in most samples and are therefore not reported here. **Supplemental Tables 2** and **3** provide summary descriptions of the nutritional importance and potential health impacts of the mineral micronutrients and toxic metals monitored in urine and/or plasma.

Analyses of mineral micronutrients and toxic metals in urine and/or plasma were carried out by the Health & Safety Laboratory of the UK Health and Safety Executive (HSE) (www.hsl.gov.uk; Buxton, United Kingdom). Analyses of total phenolics and salicylic acid concentrations in urine were done in the Human Nutrition Research Centre laboratory at Newcastle University using standard protocols. Total phenolic excretion was assessed by the Folin-Ciocalteu method described by Zhang et al. (30), which is considered a valid nutritional biomarker for total dietary (poly)phenolic intake and a proxy biomarker of dietary fruit and vegetable intake (31). Salicylic acid excretion was assessed by HPLC with Coularray detection using the method described by Baxter et al. (32). UE of salicylic acid was reported to be a suitable biomarker for plant-derived dietary salicylates intake, which is not affected by the use of aspirin (33).

### Study outcomes

The primary outcome was UE of mineral micronutrients, toxic metals, and phenolics and plasma concentrations of selected mineral micronutrients (Cu, Fe, Se, Zn). The study was registered at clinicaltrials.gov before enrolment of the study participants. At the time of data analyses (i.e., when the trial was completed) we were able to include additional elements compared with those registered, e.g., Zn. We chose this primary outcome because both MedDiet and organic food consumption were reported to lead to higher intakes of phenolics (the most abundant secondary metabolites/antioxidants in plants) and mineral micronutrients, but lower exposure to the toxic metal Cd, which in turn has been linked to positive effects on human health (1–3, 13, 14, 17–25, 34, 35). We included salicylic acid as an additional phenolic biomarker, because salicylic acid and its natural derivatives (salicylates) have been linked to a wider range of health-relevant impacts including anti-inflammatory, anticancer, neuroprotective, and antidiabetic effects (35).

We also included a range of mineral micronutrients and toxic metals which were not previously compared in organic and conventional foods (17–27) to enable effects of diet and food type to be determined for a more comprehensive range of essential mineral micronutrients and toxic metals. UE was previously described as a suitable biomarker for measuring differences in exposure to or dietary intake of Cd, Co, Mo, Ni, Pb, and Se (36–38). Plasma concentrations were determined for selected essential mineral micronutrients (Cu, Fe, Se, Zn), for which *1*) insufficient dietary intake is a public health concern globally (Fe, Se, Zn) and/or *2*) UE has been reported not to be an accurate biomarker for nutrient intake (Cu, Fe, Zn) (36–38).

We did not monitor phytochemicals other than phenolics, mainly because *1*) the effects of food type reported were relatively low (on average 10% higher concentrations in organic than conventional crops) (17, 18, 25) and *2*) the effects of diet on the intake of these phytochemicals are already well documented (1–4). We chose not to monitor fatty acid profiles, because *1*) MedDiets are well documented to change dietary fatty acid intake [e.g., increase oleic acid (18:1n–9) intake owing to higher olive oil consumption] (3–5) and *2*) significant effects of food type on concentrations of nutritionally relevant fatty acids in the context of a MedDiet were unlikely to be large enough to be detected. Most importantly, during the intervention period *1*) both groups consumed the same fish [1 main dietary source for the nutritionally desirable very-long-chain ω-3 fatty acids EPA (20:5n–3), docosapentaenoic acid (DPA; 22:5n–3), and DHA (22:6n–3)], *2*) consumption of meat (the other main dietary source for very-long-chain ω-3 fatty acids) with the MedDiet was substantially lower than with the habitual Western Diets, and *3*) most of the dairy and meat products consumed were from organic and conventional small ruminant production systems in Crete, which use similar extensive or semi-intensive grazing-based feeding regimes and therefore produce products with similar fatty acid profiles (39).

However, we assessed diet composition (Table 1) (28) to *1*) allow the relative importance of different diet components on phenolic, mineral micronutrient, and toxic metal excretion to be estimated by redundancy analysis (RDA) and *2*) identify potential confounding effects of diet composition when the effects of food type (organic or conventional) were compared during the intervention period (17, 18).

**FIGURE 1:**
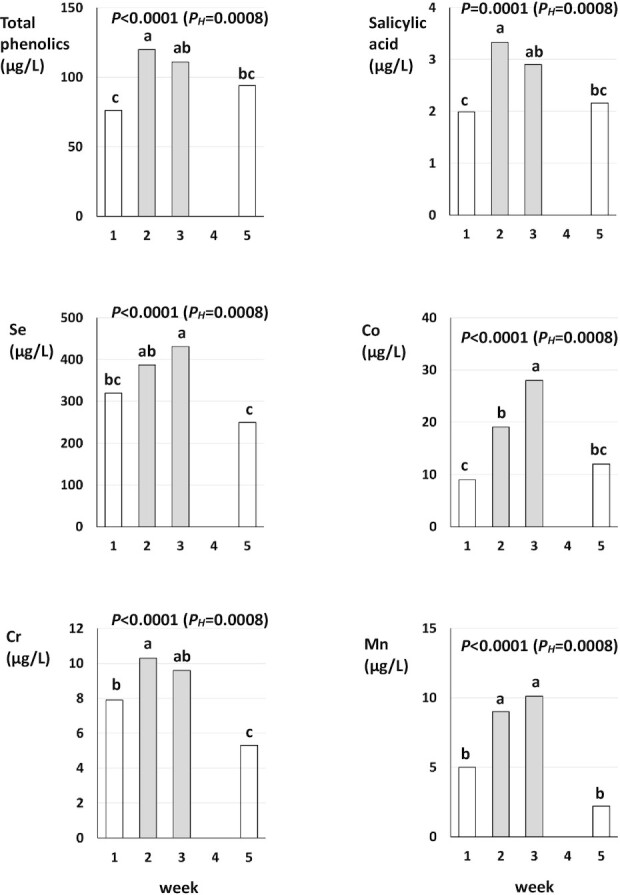
Urinaryexcretion of total phenolics, salicylic acid, selenium (Se), cobalt (Co), chromium (Cr), and manganese (Mn) in weeks 1, 2, 3, and 5 of the experiment; data shown are main effect means and *P* values for sampling week (*n* = 27) from a 3-factor ANOVA with sex, sampling week, and participant group as factors (see **Supplemental Tables 8** and **9** for 95% CIs). White bars indicate that participants consumed habitual Western Diets in the week before measurements were taken. Gray bars indicate that participants consumed a Mediterranean Diet in the week before measurements were taken. *P*, nonadjusted *P* value; *P_H_*, Holm-adjusted *P* value. Bars in each graph without a common letter are significantly different according to Tukey contrasts (general linear hypothesis test; P < 0.05).

**FIGURE 2:**
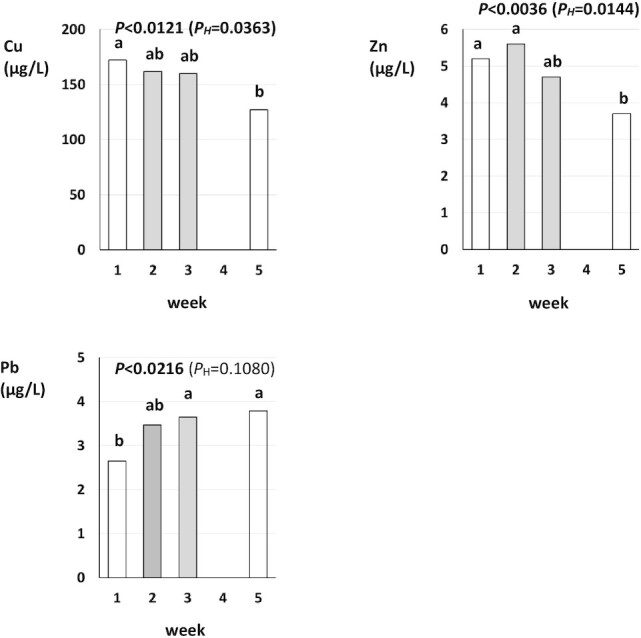
Urinary excretion of copper (Cu), zinc (Zn), and lead (Pb) in weeks 1, 2, 3, and 5 of the experiment; data shown are main effect means and *P* values for sampling week (*n* = 27) from a 3-factor ANOVA with sex, sampling week, and participant group as factors (see Supplemental Tables 8 and 9 for 95% CIs). White bars indicate that participants consumed habitual Western Diets in the week before measurements were taken. Gray bars indicate that participants consumed a Mediterranean Diet in the week before measurements were taken. *P*, nonadjusted *P* value; *P_H_*, Holm-adjusted *P* value.

To confirm that the 2-wk crossover period was sufficiently long to assess the effect of diet change we compared UE concentrations at the end of week 1 and week 2 of the intervention period (**Figures 1** and **2**).

Because cereal products are one of the main dietary sources for phenolics, minerals, and toxic metals, and virtually all cereal products consumed during the intervention period were produced and/or sourced in Germany and the United Kingdom, we also carried out a supplementary wheat flour survey in these 2 countries [results were previously published (24)]. In this survey all accessible organic and conventional, white and whole-grain flour brands were analyzed for mineral, toxic metal, and phenolic concentrations, thus allowing accurate estimates of intakes of these compounds (24).

### Statistical analyses

Because comparative studies on UE from organic and conventional food consumption were not available for the mineral and phenolic biomarkers assessed in this study, statistical power calculations were based on published pesticide residue excretion data and have been detailed previously (28). In their randomized crossover study examining the effect of a 1-wk organic food intake on pesticide UE concentrations, Bradman et al. (40) found a mean reduction in dialkylphosphates in urine from ∼150 to ∼90 nmol/L when switching from a conventional to an organic diet. To achieve such a marked difference with a power of 80%, a type I error of 5%, and assuming an SD of 45, we needed in total 16 participants. However, because compliance can be a challenge in this type of intervention study, and because relative differences in phenolic and mineral concentrations between organic and conventional crops were reported to be substantially lower than those reported for pesticides (17, 24), we included 27 participants (28).

We used mixed-effect models, with participant as a random effect, to assess impacts of the covariates as fixed effects on the excretion of metabolites and food consumption, removing nonsignificant covariates and comparing models using ANOVA (41). Covariates/factors assessed were sex (female, male), sampling week, participant group (conventional, intervention), diet (habitual Western, Mediterranean), and/or food type (organic, conventional) using the “*nlme*” package in R (www.r-project.org; 42). Sex was included as a factor because previous studies reported differences in absorption and excretion of some toxic metals (e.g., Al) and mineral micronutrients (e.g., Se) between sexes (43, 44). The normality of the model residuals was tested using quantile-quantile (QQ)-plots (“*qqnorm*” function in R). Significant interactions were further investigated by comparing interaction means using Tukey contrasts in the general linear hypothesis testing “*glht*” function of the multcomp package in R (45). Real means and SEs of means were generated by using the “*tapply*” function, whereas 95% CIs were generated by using the “*t.test*” function in R. The proportion of variance explained by the random factor (participant) in the mixed-effects models (% of total residual) was calculated by dividing the variance of the random effect by the sum of the variance of the random effect + residuals; this was done to provide an estimate of the variation associated with physiologic/genetic differences between individuals. To assess how collinearity in predictor variables may have influenced the extent to which we could assess the contribution of each covariate to the outcomes we calculated variance inflation factors (VIFs) using the “*performance*” package in R. In addition, we calculated Holm-adjusted *P* values (*P_H_*) using the “*p.adjust*” functions in R to estimate/address the risk of potential multiple-testing errors.

Because 3-factor ANOVA with sex, sampling week (week 2, week 3), and food type (organic, conventional) did not detect significant effects of food type for any of the parameters assessed (**Supplemental Tables 4**–**7**), food type/participant group was not included as a factor in the ANOVA which assessed the effect of diet change (habitual Western Diet to MedDiet, MedDiet to habitual Western Diet).

Three-factor ANOVA with sex, sampling week (week 1, week 2, week 3, week 5), and participant group as factors identified significant differences between week 1 and week 5, and/or week 3 and week 4 for several parameters (**Supplemental Tables 8**–**13**; Figures 1 and 2). We therefore carried out separate analyses for *1*) the habitual Western Diet to MedDiet switch-over (comparing data from week 1 and week 3) and *2*) the MedDiet to habitual Western Diet switch-over (comparing data from week 3 and week 5) (**Tables 2**–**5**).

**TABLE 2:** Effects of switching from habitual Western Diets to a MedDiet and from a MedDiet to habitual Western Diets on the daily urinary excretion of phenolic markers, mineral micronutrients, and toxic metals and plasma concentrations of Cu, Fe, Se, and Zn^1^

Parameters	Habitual Western Diet in week 1 (*n* = 27)	Diet (Western Diet to MedDiet switch-over), *P* values for main effect	Mediterranean Diet in week 3 (*n* = 27)	Diet (MedDiet to Western Diet switch-over), *P* values for main effect	Habitual Western Diet in week 5 (*n* = 27)	Western Diet to MedDiet switch-over *P* values		MedDiet to Western Diet switch-over *P* values	
						Main effect, sex	Interaction, sex × diet	Main effect, sex	Interaction, sex × diet
Phenolic markers, µmol/d									
Total phenolics	76 (70, 82)	<0.0001 (0.0002)	111 (105, 118)	0.0651 (0.5859)	94 (87, 101)	0.7494	0.7078	0.2063	0.2188
Salicylic acid	2.0 (1.8, 2.2)	0.0036 (0.0396)	2.9 (2.7, 3.1)	0.0178 (0.2136)	2.2 (1.9, 2.4)	0.9726	0.0666	0.1055	0.7779
Mineral micronutrients, nmol/d (unless stated otherwise)									
Co	9 (8, 11)	0.0001 (0.0015)	28 (23, 32)	<0.0001 (0.0002)	12 (10, 14)	0.0360^2^ (0.5042)	0.0364^3^ (0.5828)	0.0293^2^ (0.4691)	0.0481^3^ (0.7701)
Cr	7.9 (7.4, 8.4)	0.0166 (0.1162)	9.6 (9.0, 10.2)	<0.0001 (0.0002)	5.3 (4.8, 5.8)	0.5832	0.8485	0.5206	0.9157
Cu	172 (157, 187)	0.4563	160 (150, 169)	0.0199 (0.2189)	127 (116, 139)	0.0311^2^ (0.4668)	0.5016	0.2472	0.4352
Plasma Cu, µmol/L	13 (12, 13)	0.3410	13 (12, 14)	0.1779	13 (13, 14)	0.0633	0.6118	0.0349^2^ (0.5238)	0.7271
Plasma Fe, µmol/L	24 (22, 26)	0.5457	26 (24, 28)	0.7361	25 (24, 27)	0.7720	0.2052	0.5385	0.6898
I, µmol/d	1.0 (0.9, 1.1)	0.0001 (0.0015)	1.7 (1.6, 1.8)	0.4365	1.4 (1.0, 1.8)	0.9040	0.5484	0.2281	0.2166
Mn	5.0 (4.3, 5.8)	0.0061 (0.0610)	10.1 (8.6, 11.6)	<0.0001 (0.0002)	2.2 (2.0, 2.4)	0.1673	*0.0524*	0.0781	0.0502
Se	319 (289, 350)	0.0120 (0.0960)	431 (401, 462)	0.0001 (0.0013)	249 (224, 274)	0.5822	0.3212	0.7021	0.9805
Plasma Se, µmol/L	1.02 (1.00, 1.05)	<0.0001 (0.0015)	1.16 (1.14, 1.19)	0.4549	1.15 (1.13, 1.17)	0.1774	0.5527	0.2346	0.8725
Zn, µmol/d	5.2 (4.7, 5.8)	0.2693	4.7 (4.2, 5.1)	0.0525	3.7 (3.2, 4.1)	0.1544	0.8791	0.0549	0.7690
Plasma Zn, µmol/L	13 (12, 13)	0.6689	13 (12, 13)	0.1110	13 (13, 13)	0.0238^2^ (0.3811)	0.6005	0.0919	0.2980
Toxic metals, nmol/d									
Al	267 (218, 316)	0.7862	285 (238, 333)	0.5044	244 (184, 305)	0.2016	0.0387^3^ (0.5829)	0.0352^2^ (0.5238)	0.5101
Ni	59 (55, 63)	0.0005 (0.0060)	84 (77, 91)	0.2553	71 (62, 80)	0.2160	0.7306	0.5611	0.6057
Pb	2.7 (2.4, 2.8)	0.0065 (0.0610)	3.7 (3.3, 4.0)	0.7700	3.8 (3.3, 4.2)	0.0731 (0.8772)	0.4045	0.1340	0.7785

1 Values are main effect means (95% CIs) unless indicated otherwise. Holm-adjusted *P* values are shown in parentheses, and the estimated proportion of variation associated with the random factor (“participant”) and variance inflation factors are presented in Table 5. MedDiet, Mediterranean Diet.

2 See Table 3 for main effect means and 95% CIs.

3 See Table 4 for interaction means ± SEs.

Separate 2-factor ANOVA with sex and diet as factors for the 2 diet switch-overs identified significant main effects of sex and/or significant interaction between sex and diet for some parameters (Al, Co, Cu, Zn) based on nonadjusted, but not Holm-adjusted, *P* values, whereas most significant main effects of diet remained significant (*P* > 0.05) after Holm-adjustment of *P* values. Because *1*) the VIFs for main effects of sex and the interaction between sex and diet were generally low (<4; Table 5), *2*) relative differences in UE between males and females were very large for some parameters (e.g., Al and Co; Table 4), and *3*) there is concern that multiple-testing adjustments may potentially “hide” significant effects, we report both nonadjusted and Holm-adjusted *P* values (Table 1, Figures 1 and 2).

**TABLE 3:** Effect of sex on urinary excretion of Al, Co, Cu, and Zn and plasma concentrations of Cu and Zn^1^

	Sex			
Parameter	Male (*n* = 28)	Female (*n* = 26)	*P*	*P_H_*
Mineral micronutrients				
Co, nmol/d (Western Diet to MedDiet)	13 (10, 16)	24 (19, 28)	0.0360	0.5042
Co, nmol/d (MedDiet to Western Diet)	13 (11, 16)	26 (22, 30)	0.0293	0.4691
Cu, nmol/d (Western Diet to MedDiet)	146 (138, 153)	187 (172, 202)	0.0311	0.4668
Plasma Cu, µmol/L (MedDiet to Western Diet)	15 (14, 16)	12 (12, 12)	0.0349	0.5238
Plasma Zn, µmol/L (Western Diet to MedDiet)	13 (13, 13)	12 (12, 12)	0.0238	0.3811
Toxic metals				
Al (nmol/d) (MedDiet to Western Diet)	182 (149, 214)	520 (362, 677)	0.0352	0.5380

1 Values are main effect means (95% CIs). MedDiet, Mediterranean Diet; *P*, nonadjusted *P* value; *P_H_*, Holm-adjusted *P* value.

**TABLE 4:** Effect of sex and diet on urinary excretion of Al and Co (nmol/d)^1^

		Factor 2: diet	
Parameter (switch-over type)	Factor 1: sex	Western	Mediterranean
Mineral micronutrients			
Co (Western Diet to MedDiet)	Female	11 ± 3^b^ (*n* = 13)	37 ± 7^a^ (*n* = 13)
	Male	8 ± 3^b^ (*n* = 14)	18 ± 5^b^ (*n* = 14)
Co (Western Diet to MedDiet)	Female	15 ± 3^b,c^ (*n* = 13)	37 ± 7^a^ (*n* = 13)
	Male	9 ± 3^c^ (*n* = 14)	18 ± 5^b^ (*n* = 14)
Toxic metals			
Al (Western Diet to MedDiet)	Female	238 ± 22^a,b^ (*n* = 13)	404 ± 79^a^ (*n* = 13)
	Male	294 ± 94^a,b^ (*n* = 14)	174 ± 37^b^ (*n* = 14)

1 Values shown are interaction means ± SEs. Interaction means for the same parameter without a common letter are significantly different, according to Tukey contrasts (general linear hypothesis test; *P <* 0.05). MedDiet, Mediterranean Diet.

**FIGURE 3:**
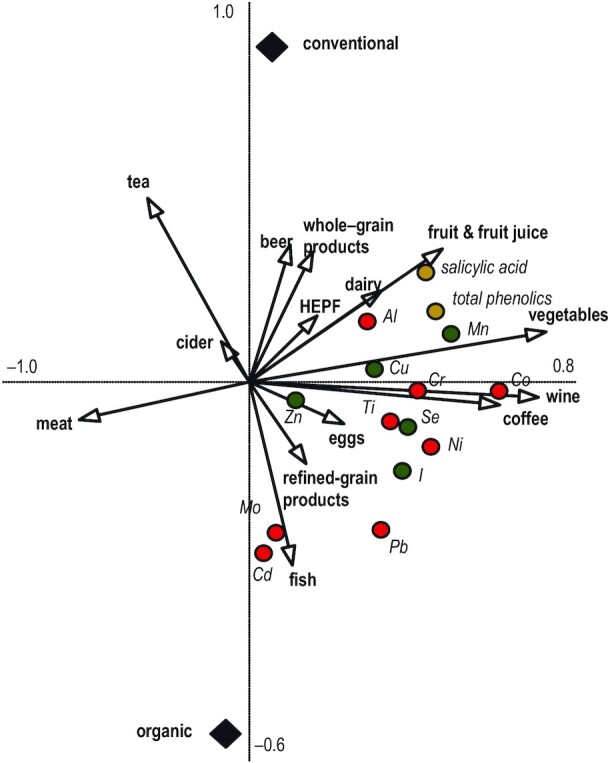
Bi-plot derived from the redundancy analysis showing the relation of food type (organic compared with conventional) and diet component explanatory variables with urinary excretion of *1*) salicylic acid and total phenolics as yellow circles, *2*) mineral micronutrients (Cu, Fe, I, Mn, Se, Zn) as green circles, and *3*) other metals (Al, Cd, Co, Cr, Mo, Ni, Pb, Ti) as red circles. Explanatory variables account for 33.6% of variation; axis 1 explains 19% and axis 2 a further 6% of variation. Continuous explanatory variables are shown as arrows and were vegetables (*F* = 10.2; *P* = 0.002), coffee (*F* = 4.0; *P* = 0.006), wine (*F* = 2.2; *P* = 0.062), fish (*F* = 2.2; *P* = 0.080), tea (*F* = 1.9; *P* = 0.088), fruits and fruit juice (*F* = 1.7; *P* = 0.124), eggs (*F* = 1.5; *P* = 0.182), whole-grain cereal products (*F* = 1.3; *P* = 0.210), dairy products (*F* = 1.2; *P* = 0.254), total meat (*F* = 1.0; *P* = 0.348), cider (*F* = 0.8; *P* = 0.572), refined-grain cereal products (*F* = 0.7; *P* = 0.63), and processed, energy-dense foods (*F* = 0.5; *P* = 0.772). Fixed explanatory variables are shown as black diamonds and were the 2 types of food consumed: conventional (*F* = 0.9; *P* = 0.49) and organic (*F* = 0.9; *P* = 0.49). HEPF, high-energy processed food.

The influence of diet components on UE of phenolic markers, mineral micronutrients, and toxic metals was assessed using partial redundancy analyses (pRDAs) in CANOCO 5 (46). The total variance of the data set shows how much variation in the response variables was redundant with the variation in the explanatory variables (47). In addition, the effect of specific explanatory factors (e.g., unwanted variation caused by individual participants in the present work) on a set of response variables was accounted for in the model by using participant as a covariable (pRDA) before a standard RDA (48). The ordination score shows how much variation in the response variables was redundant with the variation in the explanatory variables (constrained variation). In the resulting biplots (**Figure 3**), the arrows’ direction and length demonstrate the relative effects of explanatory variables (diet components) relative to the response variables (UE of phenolic markers, mineral micronutrients, and toxic metals; which are presented as points). The statistical significance of the relation between the response variables and the whole set of explanatory variables was calculated by using automatic forward selection of variables and the Monte Carlo permutation test.

## Results

### Recruitment of study participants

Twenty-seven students were recruited between 1 February and 31 March, 2017 from a total of 30 eligible students taking part in an agricultural field course in Crete (Supplemental Figure 1). Except for an unbalanced sex representation in the 2 participant groups (38% male in the intervention group and 64% male in the conventional group), there were no significant differences in mean age, weight, body fat, and height between the groups at baseline (Supplemental Table 1). No harms or unintended effects were detected in participants during the study.

### Effects of food type

No significant main effects of food type/participant group could be detected for any of the parameters assessed in urine and plasma when data from the conventional and intervention groups in week 2 and/or week 3 (= intervention period) were used to compare the effect of organic with conventional food consumption in the context of a MedDiet (Supplemental Tables 4 and 6). After Holm-adjustment of *P* values there were also no significant interactions between sex and food type, although it should be noted that the nonadjusted *P* value for the UE of salicylic acid was significant (Supplemental Table 4).

Analysis of food diary data showed that, during the intervention period, the diets of the intervention and conventional groups were very similar, except for a significantly higher (*P* = 0.032) consumption of dairy products and a trend (0.1 > *P* > 0.05) toward higher consumption of high-energy processed foods by the intervention group [results of the food diary analyses were published previously (28)].

### Effects of diet

Analysis of food diary data identified substantial differences in the consumption of different foods/diet components between the habitual Western Diets and the MedDiet (Table 1). For example, consumption of fruit/fruit juice, wine, vegetables, refined-cereal products, eggs, dairy products, and coffee were 2.6, 9.1, 1.5, 1.9, 2.3, 1.4, and 2.3 times higher, respectively, with the MedDiet than with the habitual Western Diets (Table 1). In contrast, consumption of meat and tea were 2.7 and 7.5 times higher, respectively, with the habitual Western Diets, and whole grain and beer consumption did not increase when participants changed to a MedDiet during the intervention period (Table 1). However, ANOVA detected no significant difference between the diets consumed by male and female participants, except for a 76% higher consumption of bread made from refined flour by male participants (Table 1).

Data from both participant groups were pooled when the effects of diet change (habitual Western Diet to MedDiet and MedDiet to habitual Western Diet) on UE and/or plasma concentrations of phenolics, mineral micronutrients, and toxic metals were analyzed (Tables 2–4, Supplemental Tables 8–13), because no significant effects of food type/participant group were detected during the intervention period (see “Effects of food type” and Supplemental Tables 4–7).

Changing from self-selected, habitual Western Diets to a controlled MedDiet for 2 wk (= data collected at the end of week 3 of the study) resulted in significant increases in UE of total phenolics (by 46%), salicylic acid (by 45%), the mineral micronutrients Co, Cr, I, Mn, and Se (by 211%, 22%, 70%, 102%, and 35%, respectively), and the toxic metals Ni and Pb (by 42% and 37%, respectively), and plasma Se concentrations (by 14%) (Table 2). Changing from a MedDiet to the habitual Western Diets resulted in a nominal reduction in UE/plasma concentration for the same parameters except Pb, but differences were not significant for UE of total phenolics, I, and Ni and plasma Se concentrations (Table 2).

When UE in different sampling weeks was compared, consumption of the habitual Western Diets resulted in significantly lower UE of Cr, Cu, and Zn, but higher UE of Pb, before (week 1) than after (week 5) the intervention period (Figures 1 and 2), although the Western Diets consumed in week 1 and week 5 were very similar (28). Also, except for Co, UE concentrations were not significantly different at the end of week 1 and week 2 of the intervention period (Figures 1 and 2).

It is important to consider that after Holm-adjustment of *P* values the main effects of diet on *1*) UE of Cr, Mn, Se, and Pb in the Western Diet to MedDiet switch-over and *2*) UE of total phenolics, salicylic acid, and Cu in the MedDiet to Western Diet switch-over were nonsignificant (Table 2).

### Effects of sex and variation explained by participant

Significant effects of sex were detected for the UE of Al, Co, Cu, and Zn and plasma Cu and Zn concentrations, with UE being lower and plasma concentrations higher in females (Tables 2 and 3, Supplemental Tables 4 and 7). For UE of Al and Co significant interactions between sex and diet were also detected, with significantly higher UE in females being detected after consumption of a MedDiet, but not habitual Western Diets (Table 4). It is important to note that after Holm-adjustment of *P* values all significant main effects of sex and interactions between sex and diet were nonsignificant (Table 2, Supplemental Tables 4 and 7) and that a substantial proportion (between 20% and 40% for many of the parameters assessed) of the total variation was explained by the random factor (participant) (Table 5, Supplemental Tables 6–13).

**TABLE 5:** Proportion of variance explained by the random factor participant (variance of the random effect/sum of variance of the random effect + residuals) and VIFs for the main effects and interaction between sex and diet in the mixed-effects model–based ANOVAs reported in Table 2^1^

	Western Diet to MedDiet switch-over				MedDiet to Western Diet switch-over			
		VIFs				VIFs		
	Variance explained by random factor, %	Main effects		Interaction	Variance explained by random factor, %	Main effects		Interaction
Parameters	Participant	Sex	Diet	Sex × diet	Participant	Sex	Diet	Sex × diet
Phenolic markers								
Total phenolics	57	1.2	2.1	2.3	26	1.8	2.1	2.9
Salicylic acid	41	1.5	2.1	2.6	31	1.7	2.1	2.7
Mineral micronutrients								
Co	37	1.6	2.0	2.7	52	1.3	2.0	2.3
Cr	32	1.7	2.1	2.8	0	2.0	2.1	3.1
Cu	24	1.8	2.1	2.9	35	1.6	2.1	2.7
Cu (plasma)	78	1.0	2.1	2.1	70	1.1	2.1	2.2
Fe (plasma)	23	1.9	2.1	2.9	18	1.9	2.1	3.0
I	0	2.0	2.1	3.1	30	1.7	2.1	2.8
Mn	100	2.0	2.1	3.1	24	1.8	2.1	2.9
Se	24	1.8	2.1	2.9	8	2.0	2.1	3.1
Se (plasma)	60	1.2	2.1	2.3	62	1.2	2.1	2.2
Zn	49	1.4	2.1	2.4	42	1.5	2.1	2.6
Zn (plasma)	46	1.4	2.1	2.5	33	1.7	2.1	2.8
Toxic metals								
Al	8	2.0	2.1	3.1	41	1.5	2.1	2.6
Ni	44	1.5	2.0	2.5	19	1.9	2.0	3.0
Pb	35	1.7	2.0	2.7	26	1.8	2.0	2.9

1 MedDiet, Mediterranean Diet; VIF, variance inflation factor.

### Associations of food type and diet components with UE profiles

An exploratory RDA was carried out to study associations of diet components and food type (organic compared with conventional) with UE of phenolic markers, mineral micronutrients, and toxic metals (Figure 3). In the bi-plot shown in Figure 3 the food production system and diet composition included in the RDA explained 33.6% of the variation. Vegetable consumption (*F* = 10.2; *P* = 0.002) was identified as the strongest explanatory variable/driver followed by coffee (*F* = 4.0; *P* = 0.006), wine (*F* = 2.2; *P* = 0.062), fish (*F* = 2.2; *P* = 0.080), tea (*F* = 1.9; *P* = 0.088), and fruits and fruit juice (*F* = 1.7; *P* = 0.124) excretion. Consumption of eggs, whole-grain and refined-grain cereal products, dairy, meat, cider, and high-energy processed foods and food type (organic compared with conventional) were identified as relatively weak drivers and explained only small amounts (*F* ≤ 1.5; *P* > 1.8) of the additional variation (Figure 3).

When associations between the stronger dietary drivers and UE of mineral and phenolic markers were examined, vegetables, fruits/fruit juice, wine, and coffee consumption were positively associated with the UE of the phenolic markers, all mineral micronutrients, and Al (Figure 3). In contrast, fish and to a lesser extent refined-cereal grain product consumption were more closely associated with the UE of Mo, Cd, and Pb (Figure 3).

## Discussion

This study allowed, for the first time, a detailed comparison of the effects of changing to *1*) a MedDiet with increased fruit and vegetable and lower meat consumption and *2*) organic food consumption on UE and/or plasma concentrations of phenolic markers, mineral micronutrients, and toxic metals.

Current nutritional guidelines to increase fruit and vegetable consumption from amounts reported in Western diets (1–3 portions/d) to those typical for a traditional MedDiet (≥5 portions/d) are based on a substantial body of evidence from both epidemiologic and dietary intervention studies that this change will lead to significant public health benefits (1–4). There is also increasing evidence that the health benefits of increasing fruit, vegetable, and whole grain consumption are linked, at least partially, to increased dietary intakes of phenolic compounds and minerals with apparent antioxidant capacity and anti-inflammatory activity (1–5). Our study provides further evidence that changing from a Western diet to a MedDiet with substantially higher fruit, vegetable, and wine consumption will significantly increase the intake of micronutrients for which plant foods are the main dietary source (namely, total phenolics, salicylic acid, Co, Mn, and Se). When considering potential health impacts of changing to a MedDiet, it is important to take into account that this diet not only substantially increased the UE of total phenolics (the most abundant antioxidants in plants), but also salicylic acid, a phenolic compound which has been linked to anti-inflammatory, anticancer, neuroprotective, and antidiabetic effects (33, 35).

However, diet change did not affect UE and/or plasma concentrations of mineral micronutrients (namely, Cu, Fe, and Zn) for which both plant and animal products (especially meat) can contribute significantly to dietary intakes (48). This was most likely because a higher intake of Cu and Zn with meat in the habitual Western Diet (meat consumption was nearly 3 times higher with the Western Diet than with the MedDiet) was compensated for by higher intakes of these mineral micronutrients with fruits and vegetables in the MedDiet. Our study also found evidence supporting previous studies which reported differences in mineral and toxic metal excretion between males and females, but it is important to point out that effects of sex were nonsignificant when Holm-adjustments of *P* values were carried out to avoid multiple-testing errors (43, 44).

In contrast, our study detected no significant effects of food type (organic compared with conventional) on the UE and/or plasma concentrations of the 2 phenolic markers (total phenolics and salicylic acid); a wide range of mineral micronutrients including Cu, Fe, I, Se, and Zn; and a range of toxic metals including Cd and Pb. These results are consistent with several previous dietary intervention studies which assessed the effect of consuming specific organic foods (e.g., apples, carrots, or tomatoes) in the context of a conventional food-based diet (14, 49–52).

However, several studies which assessed the effect of replacing a larger proportion of the food with organic products reported some small, but significant, effects of food type on antioxidant and micronutrient biomarkers (14, 53, 54). For example, Di Renzo et al. (54) reported that plasma concentrations of apparent antioxidants measured by the oxygen radical absorbing capacity method in healthy men aged 30–65 y significantly increased (21%) after switching for 14 d to organic food consumption in the context of a MedDiet. Also, more recently Baudry et al. (55) compared plasma micronutrient concentrations in consumers with low (<10%; *n* = 150) and high (>50%; *n* = 150) organic food consumption but similar diets. They reported significantly (10%) higher plasma concentrations of Mg, carotenoids (α-carotene, β-carotene, lutein, and zeaxanthin), and linoleic acid (18:2n–6), and significantly lower (10%) plasma concentrations of palmitoleic acid (16:1n–7) and DPA, in consumers with high organic food consumption. None of these parameters was monitored in the study reported here, which prioritized the monitoring of phenolic, mineral micronutrient, and toxic metal biomarkers. However, similar to the results of our study, Baudry et al. (55) also did not detect significant effects of food type on plasma Fe, Cu, and Cd concentrations. It is interesting to note that UE of Se, Zn, and Mo was numerically higher, whereas UE of I and Cd was numerically lower, with organic than with conventional food consumption, which is consistent with the compositional differences between organic and conventional foods (e.g., cereals and dairy products) reported in previous studies (17–24).

RDA confirmed vegetable, wine, whole grain, and fruit consumption as the main drivers for phenolic and mineral micronutrient intake. In addition, this analysis identified a strong positive association between fish consumption and UE of Cd and Pb, which is consistent with previous studies showing that seafood is a major dietary source for toxic metals in the Mediterranean and other regions (56–58).

When considering potential health implications of the results reported here, it is important to take into account that *1*) changing from a Western Diet to a MedDiet was also shown to result in a 3- to 4-fold increase in UE of insecticides and organophosphates in the same dietary intervention trial, whereas *2*) changing from conventional to organic food consumption resulted in a >90% reduction in pesticide UE in the context of a MedDiet (28). This supports the hypotheses that *1*) lower pesticide exposure is the main driver for the positive health impacts linked to organic food consumption in observational studies (6–12, 28) and *2*) pesticide residues in fruit, vegetables, and whole-grain cereals reduce the overall beneficial health impacts of increasing whole grain, fruit, and vegetable consumption (e.g., lower levels of ischemic heart disease) (28, 59, 60).

### Study limitations

The low number of participants is the main limitation of this study. Our statistical power calculation was based on the relatively large differences (>50%) in urinary organophosphate excretion in cohorts consuming organic as opposed to conventional food reported previously (28). Given that the reported differences in phenolic and mineral concentrations between organic and conventional crops (17, 18, 21–24) were much lower (15%–20%), small, nonsignificant differences in diet composition (e.g., the 15%–20% higher fruit and whole-grain cereal consumption by the conventional group during the intervention period) may have confounded the effect of organic food consumption. Similarly, the numerically higher consumption of fish and significantly higher consumption of dairy products (and associated Cd and I intakes, respectively) by the intervention group during the intervention period (28) may have compensated for lower Cd concentrations in organic plant foods and lower I concentrations in organic dairy products. The different environments in which the Western Diet and MedDiet were consumed and associated differences in food supply chains (habitual Western Diets were consumed in the United Kingdom, whereas the MedDiet was consumed in Crete, Greece) may also have confounded the differences observed between diets and food types.

### Conclusion

The main conclusion from this study is that changing from a Western diet to a MedDiet had a substantially larger effect on phenolic and mineral micronutrient intake than switching from a conventional MedDiet to an organic MedDiet food pattern. Similar studies with a larger number of participants would be required in order to improve statistical power to detect and quantify significant effects of food type on micronutrient intake.

## Supplementary Material

nqac233_Supplemental_File

## Data Availability

All data reported in this article will be made available by the first author (Leonidas Rempelos) upon reasonable request.

## Notes

The study was funded by the Sheepdrove Trust, Drove Farm, Sheepdrove, Lambourn, Hungerford, RG17 7UN, UK (charity number:328369; www.charitychoice.co.uk/the-sheepdrove-trust-124094); the funder had no role in study design, implementation, analysis and interpretation of the data, and writing of the report.

Author disclosures: CL owns farmland in Greece that is managed to organic farming standards and farmland in Germany that is managed to conventional farming standards and is a member of the UK Soil Association. All other authors report no conflicts of interest.

Supplemental Figures 1 and 2 and Supplemental Tables 1–13 are available from the “Supplementary data” link in the online posting of the article and from the same link in the online table of contents at https://academic.oup.com/ajcn/.

Abbreviations used: DPA, docosapentaenoic acid; EU, European Union; MedDiet, Mediterranean Diet; pRDA, partial redundancy analysis; RDA, redundancy analysis; UE, urinary excretion; VIF, variance inflation factor.
