# Supplementary material for: Diet, but not food type, significantly affects micronutrient and toxic metal profiles in urine and/or plasma; a randomized, controlled intervention trial
Source: Am J Clin Nutr. 2022 Aug 30;116(5):1278–90. doi: 10.1093/ajcn/nqac233 (PMC9630859; doi:10.1093/ajcn/nqac233)
Supplement: nqac233_Supplemental_File [file nqac233_supplemental_file.docx]

**On-line Supplementary material for:**

**Diet, but not food type significantly affects micronutrient and toxic metal profiles in urine and/or plasma; a randomized, controlled dietary intervention trial**

*Leonidas Rempelos,^1,2^* Juan Wang,^2,3,4^, Marcin Barański,^2,5^ Anthony Watson,^3^ Nikolaos Volakakis,^6^ Catherine Hadall^7^, Gultakin Hasanaliyeva,^2,8^ Eleni Chatzidimitriou,^2,9^ Amelia Magistrali,^2^ Hannah Davis, ^2^ Vanessa Vigar,^10^ Dominika Średnicka-Tober,^11^ Steven Rushton,^12^ Kristin S. Rosnes,^13^ Per Ole Iversen,^13,14^, Chris J. Seal,^3^ and Carlo Leifert^10,13^**

^1^ Lincoln Institute for Agri-Food Technology, University of Lincoln, Riseholme Park, Lincoln,

Lincolnshire. LN2 2LG, UK.

^2^ School of Agriculture, Food and Rural Development, NEFG, Newcastle University, Newcastle

upon Tyne, NE1 7RU, UK.

^3^ Human Nutrition Research Centre, Population and Health Sciences Institute, Newcastle

University, Newcastle upon Tyne NE2 4HH, UK.

^4^ School of Agriculture and Biology, Shanghai Jiao Tong University, China.

^5^ Laboratory of Neurobiology, Nencki Institute of Experimental Biology, Polish Academy of

Sciences, Pasteura 3, Warsaw 02-093, Poland.

^6^ Geokomi plc, P. O. Box 21, Sivas-Faistos, Crete GR 70200, Greece.

^7^ Newcastle upon Tyne Hospitals, Royal Victory Infirmary, Newcastle upon Tyne, UK.

^8^ Department of Sustainable Crop and Food Protection, Faculty of Agriculture, Food and

Environmental Sciences, Universita Catollica del Sacro Cuore, I-29122 Piacenza, Italy.

^9^ French Agency for Food Environmental and Occupational Health and Safety, France (ANSES);

14 rue Pierre et Marie Curie– 94701 Maisons-Alfort, France.

^10^ Southern Cross University, Military Rd., Lismore, NSW, Australia.

^11^ Institute of Human Nutrition Sciences, Warsaw University of Life Sciences, Nowoursynowska

159c, 02-776 Warsaw, Poland.

* Corresponding authors: Leonidas Rempelos, [lrempelos@lincoln.ac.uk](mailto:lrempelos@lincoln.ac.uk); Carlo Leifert, [carlo.leifert@scu.edu.au](mailto:carlo.leifert@scu.edu.au);

**Table of Contents**

**Page**

**Supplementary Table 1.** Study population at baseline 4

**Supplementary Table 2.** Summary of the nutritional importance of the mineral 5

micronutrients for which significant effects or trends towards significant effects of diet

or food type on urinary excretion were detected; unless stated otherwise estimated daily

intakes (EDI) are for Europe and adequate daily intakes (AI) and tolerable upper daily

intake levels (UL) were set by EFSA.

**Supplementary Table 3.** Summary of the toxicity and potential health impacts of toxic 7

metals (Al, Ni, Cd, Pb) for which significant effects or trends towards significant effects

of diet or food type on urinary excretion were detected (unless stated otherwise estimated

daily intakes (EDI) are for Europe and tolerable upper daily intakes (TDI) were set by

EFSA).

**Supplementary Table 4.** Effects of sex (male, female), sampling week (Wk 2, Wk3) 9

And food type (conventional, organic) on the daily urinary excretion of phenolics

salicylic acid, toxic metals (Al, Ba, Cd, Pb, Sn) and titanium (Ti).

**Supplementary Table 5.** Effect of sex and food type/participant group on urinary 11

excretion of salicylic acid (µg/day).

**Supplementary Table 6.** Effects of sex (male, female), sampling week (Wk 2, Wk3) 12

and food type (conventional, organic) on the daily urinary excretion (nmol/day unless

stated otherwise) of mineral micronutrients (Co, Cr, Cu, I, Mn, Mo, Se, Zn).

**Supplementary Table 7.** Effects of sex (male, female) and food type (conventional, 14

organic) on plasma concentrations of selected mineral micronutrients (Cu, Fe, Se, Zn)

in week 3 of the experiment.

**Supplementary Table 8.** Effects of sex (male, female), sampling week and participant 15

group (intervention, conventional) on the daily urinary excretion of phenolics salicylic

acid, toxic metals (Al, Ba, Cd, Pb, Sn) and titanium (Ti).

**Supplementary Table 9.** Effects of sex (male, female), sampling week and participant 17

group (intervention, conventional) on the daily urinary excretion **(**nmol/day unless stated

otherwise) of mineral micronutrients (Co, Cr, Cu, I, Mn, Mo, Se, Zn).

**Supplementary Table 10.** Effect of sex, sampling week and study participant urinary 19

excretion of salicylic acid (µmol/day).

**Supplementary Table 11.** Effect of sex and sampling week on urinary excretion of 20

cobalt (Co) (nmol/day).

**Supplementary Table 12.** Effect of sex and participant group on urinary excretion of 21

selenium (Se) (nmol/day).

**Supplementary Table 13.** Effects of sex (male, female), sampling week and participant 22

group (intervention, conventional) on plasma concentrations of selected mineral

micronutrients (Cu, Fe, Se, Zn) in weeks 1, 3 and 5 of the experiment.

**Supplementary Figure 1.** Flow diagram of the study design based on CONSORT 23

2010 guidelines.

**Supplementary Figure 2.** Study design; diet and food types (organic vs conventional) 24

consumed and assessments carried out before, during and after the intervention period.

| **Supplementary Table 1.** Study population characteristics at baseline. | | | |
| --- | --- | --- | --- |
|  |  | **Intervention**  **group (n=13)** | **Conventional**  **group (n=14)** |
| **Sex n** (%) |  |  |  |
| Male |  | 5 (38%) | 9 (64%) |
| Female |  | 8 (62%) | 5 (36%) |
|  |  |  |  |
| **Ethnicity^1^ n** (%) |  |  |  |
| White |  | 6 (46%) | 5 (36%) |
| Asian |  | 3 (23%) | 4 (29%) |
| Black |  | 4 (31%) | 5 (36%) |
|  |  |  |  |
| **Smokers n** (%) |  | 1 (8%) | 2 (14%) |
|  |  |  |  |
| **Weight** (kg, mean (range)) |  | 64 (49-82) | 69 (38-95) |
| Male |  | 72 (57-82) | 72 (56-94) |
| Female |  | 58 (48-73) | 63 (38-84) |
|  |  |  |  |
| **Body fat** (%, mean (range)) |  | 21 (11-31) | 19 (10-31) |
| Male |  | 17 (11-26) | 15 (10-21) |
| Female |  | 24 (12-31) | 27 (23-31) |
|  |  |  |  |
| **Height** (cm, mean (range)) |  | 169 (155-184) | 173 (149-190) |
| Male |  | 177 (163-184) | 178 (169-190) |
| Female |  | 165 (155-174) | 164 (149-175) |
|  |  |  |  |
| **Age at inclusion** (years, mean (range)) |  | 27 (21-31) | 28 (22-36) |
| Male |  | 29 (24-31) | 29 (22-36) |
| Female |  | 26 (21-30) | 25 (23-30) |
| **^1^**, self-reported by participants | | | |

| **Supplementary Table 2.** Summary of the nutritional importance of the mineral micronutrients for which significant effects or trends towards significant effects of diet or food type on urinary excretion were detected; unless stated otherwise estimated daily intakes (EDI) are for Europe and adequate daily intakes (AI) and tolerable upper daily intake levels (UL) were set by EFSA. | | | | | | |
| --- | --- | --- | --- | --- | --- | --- |
| **Mineral**  **Nutrient** | **EDI** | **AI** | **UL** | **Nutritional importance and deficiency symptoms** | **Toxicity and potential health risks** | **References** |
| Co (µg) | 5-40 | ND | 1,600  (HBGV) | Co(III) is a component of cobalamin (VitB_12_).  Co(II) is used as a feed additive in livestock.  ADI for cobalamin is 4 µg**^3^** | Cobalt(II) cations have CMR properties.  HBGV is more than 40 times higher than the EDI | EFSA 2012  EFSA 2015 |
| Cr (µg) | 25-160 | 30-100^4^ | 21,000 | Cr(III) was shown to affect insulin action.  Cr(III) can be used as a food additive.  Cr-deficiency has not been observed in healthy humans.  Cr-deficient rats show glucose intolerance similar to diabetes | Cr(III) exposure resulted in some reproductive and geno-toxicity *in vitro* and/or animal studies  UL is more than 100 times higher than the EDI and AI | EFSA 2006  EFSA 2014a |
| Cu (mg) | 0.9-2.3 | 1.1 | 5.0 | Cu is an essential component in enzymes/non-enzymatic proteins  Cu is required for infant growth, host defense mechanisms, bone strength, iron transport, cholesterol and glucose metabolism  Cu-deficiency is rarely observed in healthy humans | Acute or chronic copper toxicity in humans is rare**^8^**  High Cu exposure causes liver damage**^8^**  UL is 2-5 times higher than the EDI  and **⁓**4.5 times higher than the AI | EFSA 2006 |
| I (µg) | 141-300**^5^**  59-114**^6^** | 150**^5,6^**  200**^7^** | 1100**^5^**  600**^6^** | I is an essential component of thyroid hormones  I is used as a feed additive in livestock  Deficiency leads to I insufficient thyroid function  **EDI is significantly lower than ADI in Europe** | Excessive I-intake causes hyperthyroidism and was linked to autoimmune thyroiditis  UL is 4-10 times higher than the EDI and  **only** ⁓**3 times higher than the AI** | EU 2002  WHO 2007  EFSA 2014b |
| Mn (mg) | 2-9 | 3 | ND | Mn is an essential component of certain enzymes  No deficiency syndrome is described for humans | Mn has a relatively low acute toxicity.  Mn levels linked to neurotoxic effects are close to the EDI. | EFSA 2006  EFSA 2013 |
| Mo (µg) | 58-250 | 65 | 600 | Mo is an essential component of certain enzymes  Mo deficiency has not been observed in healthy individuals | High dietary Mo intake was linked to high serum uric acid levels and gout-like symptoms  UL is 2.5-10 times higher than the EDI and  **⁓**9 times higher than the AI | EFSA 2006  EFSA 2013  EFSA 2018 |
| Se (µg) | 24-110 | 55 | 300 | Se is an essential component of certain enzymes  Low Se-status has been linked to higher cancer and cardiovascular disease and effects on immune function  **EDI is lower than the ADI for a proportion of Europeans** | Se (including forms used in supplements) has a relatively high acute toxicity.  UL is 2.8-12 times higher than the EDI and  ⁓5.5 times higher than the AI | EFSA 2006  EFSA 2018 |
| Zn (mg) | 7.5-12.1 | 11.0**_m_^5^**  8.0 **_f_^5^**  5.5**_m_^6^**  7.3**_f_ ^6^** | 25 | Zn is an essential component in enzymes/non-enzymatic proteins  Zn is used as a feed additive in livestock  Dietary requirement for Zn is 50% greater for vegetarians  **Zn-deficiency is a significant public health issue globally** | High Zn intake can affect Cu balance and Fe storage and can lead to Cu deficiency symptoms  UL is 2-3.5 times higher than the EDI  **only ⁓4 times higher than the AI** | EFSA 2006  EFSA 2018 |

| **Legend for Supplementary Table 2.**  **EDI,** estimated daily intakes**; AI**, adequate daily intake; UL, tolerable upper intake level (UL) for an adult with 70 kg body weight; **_m_**, male; **_f_**, female; **CMR**, carcinogen, mutagen and reproduction toxicant; ND, no TDI determined/set by EFSA; **HBGV**, health-based guidance value; **^1^** EFSA 2018; **^2^**, value is the health-based guidance value (EFSA 2012); **^3^**, EFSA 2006; **^4^**, ADI suggested by the Societies for Nutrition of Germany, Austria and Switzerland; EFSA considers there to be currently insufficient evidence for the essentiality of Cr(III) in humans and has not set an AI (EFSA 2006&2014); **^5^** adults in the USA (values shown are recommended daily allowances, RDAs); **^6^,** adults in Europe (values shown are population reference values, PRAs); **^7^**, pregnant woman in Europe (EFSA 2014); **^8^**, usually linked to contamination events and professional exposure, including handling of Cu-based pesticides; Co, Cobalt; Cr, chromium; I, iodine; Mn, manganese; Mo, molybdenum; Se, selenium; Zn; zinc. |
| --- |

| **Supplementary Table 3.** Summary of the toxicity and potential health impacts of toxic metals (Al, Ni, Cd, Pb) for which significant effects or trends towards significant effects of diet or food type on urinary excretion were detected (unless stated otherwise estimated daily intakes (EDI) are for Europe and tolerable upper daily intakes (TDI) were set by EFSA). | | | | |
| --- | --- | --- | --- | --- |
| **Toxic** |  |  |  |  |
| **metals** | **EDI** | **TDI** | **Health impacts** | **References** |
| Al (µg) | 2,000-13,000 | 10,000 | Al was linked to neurotoxicity in patients undergoing dialysis and increased risk of neurodegenerative diseases.  The TDI set by EFSA is likely to be exceeded in a significant part of the European population. | EFSA 2008 |
| Ni (µg) | 110-1020 | 910 | Ni is considered a human carcinogen by inhalation. Orally ingested Ni salts can cause adverse effects on kidneys, spleen, lungs and the myeloid system in experimental animals, but has not so far not shown to be carcinogenic.  EDIs of ⁓900 μg have been recorded with high intakes of foods with high Ni content (e.g. legumes, nuts and oilseeds  [~ 2 mg/kg], certain types of chocolate [3.8 mg/kg], and cocoa beans and cocoa products’ [9.5 mg/kg]).  Ni-intakes as low as 500 μg/day have been reported to aggravate hand eczema in Ni-sensitised subjects.  The EFSA CONTAM Panel concluded that Ni intakes at TDI levels, Ni-sensitized individuals may develop eczematous flare-up skin reactions | EFSA 2008  EFSA 2020 |
| Cd (µg) | 19-39 | 25 | Cd exposure in the general population is mainly from food, although smoking also contributes significantly.  Cd is retained in the kidney and liver with a very long biological half-life ranging from 10 to 30 years.  Cd primarily toxic to the kidney and may cause renal dysfunction, but also causes bone demineralisation  The IARC has classified cadmium as a human carcinogen (Group 1) and recent data on human exposure to cadmium in the general population showed significant positive associations between Cd exposure and an increased risk of lung,  endometrium, bladder, and breast cancer.  Due to their high consumption of cereals, nuts, oilseeds and pulses, vegetarians have a higher dietary Cd intake.  Smoking and regular consumption of shellfish and wild mushrooms also significantly increase Cd exposure.  The EFSACONTAM Panel recommended current exposure to Cd at the population level should be reduced. | EFSA 2009 |
| Pb (µg) | 25-170 | (250)**^1^** | Pb exposure is mainly from food, but dust and soil can be important non-dietary sources in children.  Due to its long half-life in the body, chronic toxicity of lead is of most concern for human health.  Chronic Pb-exposure leads to neurotoxicity, nephrotoxicity and cardiovascular effects children and adults.  Relatively low blood-Pb levels were negatively associated with Intelligence Quotient (IQ) score in children and systolic blood pressure and chronic liver disease in adults.  Children are most at risk since Pb is absorbed more in children than in adults and accumulates in soft tissues and bones.  Half-lives of Pb in blood and bone are approximately 30 days and 10-30 years, respectively  The main dietary source for Pb exposure are cereals, but potatoes, leafy vegetables and tap water also contribute.  The EFSA CONTAM Panel concluded that TDI of 250 μg set by the Joint FAO/WHO Expert Committee on Food Additives is no longer appropriate as there is no evidence for a threshold for critical lead-induced effects. | EFSA 2010 |

| **Legend for Supplementary Table 3.**  **EDI,** estimated daily intakes**; TDI**, Tolerable upper daily intake level for an adult with 70 kg body weight; **IARC,** International Agency for Research on Cancer; ND, no TDI determined/set by EFSA; **^1^**, provisional tolerable weekly intake (PTWI) set by the Joint FAO/WHO Expert Committee on Food Additives (EFSA 2010); Al, aluminum; Cd, cadmium; Ni, nickel; Pb, Lead. |
| --- |

| **Supplementary Table 4.** Effects of sex (male, female), sampling week (Wk 2, Wk3) and food type (conventional, organic) on the daily urinary excretion of phenolics salicylic acid, toxic metals (Al, Ba, Cd, Pb, Sn) and titanium (Ti). Values shown are **main effect means** (95% CI). | | | | | | | | | | |
| --- | --- | --- | --- | --- | --- | --- | --- | --- | --- | --- |
|  | **Phenolic markers** (µmol/d) | |  | **Toxic metals** (nmol/d) | | | | |  |  |
| **Factor** | **Total**  **phenolics** | **Salicylic**  **acid** |  | **Al** | **Cd** | **Ni** | **Pb** | **Sn** |  | **Ti**  (nmol/d) |
| **Sex** |  |  |  |  |  |  |  |  |  |  |
| Female (n=26) | **119** (112,126) | **3.4** (3.0,3.7) |  | **361** (310,413) | **2.7** (2.5,2.9) | **92** (84,99) | **4.1** (3.8,4.5) | **9.9** (6.7,13.0) |  | **1.6** (1.5,1.7) |
| Male (n=28) | **112** (105,118) | **2.9** (2.6,3.1) |  | **178** (154,202) | **2.2** (2.1,2.4) | **81** (74,88) | **3.0** (2.8,3.2) | **4.9** (3.6,6.2) |  | **1.3** (1.2,1.4) |
| **Sampling week** |  |  |  |  |  |  |  |  |  |  |
| Wk 2 (n=27) | **120** (113,126) | **3.3** (3.0,3.7) |  | **247** (209,285) | **2.7** (2.5,2.8) | **88** (80,96) | **3.5** (3.2,3.8) | **4.7** (3.7,5.7) |  | **1.6** (1.4,1.7) |
| Wk 3 (n=27) | **111** (105,118) | **2.9** (2.7,3.1) |  | **285** (238,333) | **2.2** (2.1,2.4) | **84** (77,91) | **3.7** (3.3,4.0) | **11.2** (7.4,14.9) |  | **1.4** (1.3,1.5) |
| **Food type** |  |  |  |  |  |  |  |  |  |  |
| Conventional (n=28) | **117** (110,124) | **3.0** (2.8,3.3) |  | **258** (214,301) | **2.6** (2.4,2.8) | **85** (77,92) | **3.2** (2.9,3.5) | **5.6** (4.2,7.1) |  | **1.6** (1.5,1.7) |
| Organic (n=26) | **113** (107,119) | **3.2** (2.9,3.5) |  | **275** (233,318) | **2.3** (2.1,2.4) | **88** (80,95) | **4.0** (3.6,4.3) | **8.9** (6.0,11.9) |  | **1.4** (1.3,1.5) |
| **ANOVA** *P*-values [VIF] |  |  |  |  |  |  |  |  |  |  |
| ***Main effects*** |  |  |  |  |  |  |  |  |  |  |
| Sex | 0.5335 [2.8] | 0.3127 [2.9] |  | **0.0173** [2.7] | 0.1068 [3.1] | 0.4376 [2.4] | ***0.0508*** [2.6] | 0.1791 [4.3] |  | 0.1636 [2.9] |
| Sampling Week (SW) | 0.2477 [5.4] | 0.1810 [5.4] |  | 0.3502 [5.4] | **0.0265** [5.2] | 0.3688 [5.2] | 0.5682 [5.2] | 0.1944 [5.7] |  | 0.1877 [5.3] |
| Food type (FT) | 0.6184 [3.0] | 0.9329 [3.0] |  | 0.6694 [2.9] | 0.1118 [3.2] | 0.9453 [2.5] | 0.3789 [2.8] | 0.5577 [4.2] |  | 0.1260 [3.0] |
| ***Interactions*** |  |  |  |  |  |  |  |  |  |  |
| Sex x SW | 0.4189 [5.1] | 0.9370 [5.2] |  | 0.2639 [5.0] | 0.4830 [5.2] | 0.6709 [4.6] | 0.7871 [4.8] | 0.4091 [5.8] |  | 0.8774 [5.0] |
| Sex x FT | 0.9121 [3.5] | **0.0475^1^ [**3.6] |  | 0.8512 [3.4] | 0.8085 [3.9] | 0.5122 [3.0] | 0.9456 [3.3] | 0.9448 [5.2] |  | 0.5407 [3.6] |
| SW x FT | 0.2114 [5.0] | 0.4463 [5.1] |  | 0.6834 [4.9] | 0.7138 [5.3] | 0.8875 [4.6] | 0.1916 [4.8] | 0.2168 [6.7] |  | 0.8660 [5.1] |
| Sex x SW x FT | 0.5121 [4.1] | 0.1131 [4.2] |  | 0.8509 [4.0] | 0.3586 [4.5] | ***0.0687*** [3.7] | 0.4200 [3.9] | 0.7757 [5.6 |  | 0.9468 [4.2] |
| % variation explained by participant ID | 48 | 47 |  | 51 | 41 | 62 | 54 | 0.02 |  | 47 |
| **Simplified models** |  |  |  |  |  |  |  |  |  |  |
| **1-factor ANOVA** |  |  |  |  |  |  |  |  |  |  |
| Main effect Sex |  |  |  | 0.0173 |  |  |  |  |  |  |
| Main effect SW |  |  |  |  | 0.0233 |  |  |  |  |  |
| **2-factor ANOVA** |  |  |  |  |  |  |  |  |  |  |
| Sex |  | NS |  |  |  |  |  |  |  |  |
| Food type |  | NS |  |  |  |  |  |  |  |  |
| Sex x FT |  | 0.0475 |  |  |  |  |  |  |  |  |
| **Legend for Supplementary Table 4.**  VIF, value inflation factors derived from the multicollinearity check (p.lme) in R (a VIF value <5 indicates low correlation, a value between 5 and 10 represents moderate correlation, while a VIF value larger than 10 is a sign for high non-tolerable correlation of model predictors. **^1^** see Supplementary Table 5 for interaction means ±SE | | | | | | | | | | |

| **Supplementary Table 5.** Effect of sex and food type/participant group on urinary excretion of salicylic acid (µg/day). Values shown are **interaction means** ±SE**^1^** | | | | |
| --- | --- | --- | --- | --- |
|  |  |  | **Factor 2. Food type** (participant group) | |
| **Parameter** | **Factor 1 Sex** |  | **Conventional**  (conventional) | **Organic**  (intervention) |
| **Salicylic acid** | Female |  | 2.7 ±0.5 **ab**  (n=10) | 3.8 ±0.4 **a**  (n=16) |
|  | Male |  | 3.2 ±0.3 **ab**  (n=18) | 2.2 ±0.4 **b**  (n=10) |
| Interaction means followed by the same lower-case letter are not significantly different, according to Tukey contrasts (general linear hypothesis test; *P <* 0.05). **^1^**, see Supplementary Table 5 for main effect means and details of the ANOVA which assessed effect of and interactions with food type | | | | |

| **Supplementary Table 6.** Effects of sex (male, female), sampling week (Wk 2, Wk3) and food type (conventional, organic) on the daily urinary excretion **(**nmol/day unless stated otherwise) of mineral micronutrients (Co, Cr, Cu, I, Mn, Mo, Se, Zn). Values shown are **main effect means** (95% CI). | | | | | | | | |
| --- | --- | --- | --- | --- | --- | --- | --- | --- |
| **Factor** | **Co** | **Cr** | **Cu** | **I** (µmol/d) | **Mn** | **Mo** | **Se** | **Zn** (µmol/d) |
| **Sex** |  |  |  |  |  |  |  |  |
| Female (n= 26) | **30** (26,34) | **10.3** (9.6,11.0) | **172** (162,183) | **1.6** (1.5,1.7) | **11.8** (10.1,13.6) | **662** (584,739) | **407** (383,431) | **4.5** (4.2,4.7) |
| Male (n= 28) | **17** (14,20) | **9.6** (9.0,10.2) | **150** (142,158) | **1.5** (1.4,1.6) | **7.4** (6.4,8.4) | **621** (573,669) | **412** (385,439) | **5.7** (5.2,6.3) |
| **Sampling week** |  |  |  |  |  |  |  |  |
| Wk 2 (n=27) | **19** (16,22) | **10.3** (9.6,11.0) | **162** (153,171) | **1.4** (1.3,1.5) | **9.0** (7.6,10.5) | **754** (686,822) | **387** (368,406) | **5.6** (5.1,6.0) |
| Wk 3 (n=27) | **28** (23,32) | **9.6** (9.0,10.2) | **160** (150,169) | **1.7** (1.6,1.8) | **10.1** (8.6,11.6) | **527** (478,576) | **432** (401,462) | **4.7** (4.2,5.1) |
| **Food type** |  |  |  |  |  |  |  |  |
| Conventional (n=28) | **25** (21,29) | **9.8** (9.1,10.4) | **159** (150,169) | **1.7** (1.6,1.8) | **9.8** (8.2,11.5) | **569** (518,620) | **376** (354,398) | **4.9** (4.5,5.3) |
| Organic (n=26) | **22** (18,25) | **10.1** (9.5,10.8) | **162** (154,170) | **1.4** (1.3,1.5) | **9.2** (8.0,10.5) | **717** (644,790) | **446** (418, 473) | **5.4** (4.9,5.9) |
|  |  |  |  |  |  |  |  |  |
| **ANOVA** *P*-values [VIF] |  |  |  |  |  |  |  |  |
| ***Main effects*** |  |  |  |  |  |  |  |  |
| Sex | 0.0643 [2.3] | 0.5228 [3.2] | 0.1974 [2.5] | 0.6323 [2.6] | 0.1048 [2.7] | 0.6959 [2.9] | 0.9073 [2.6] | 0.1016 [2.8] |
| Sampling Week (SW) | **0.0009** [5.2] | 0.3770 [5.4] | 0.7377 [5.4] | **0.0063** [5.4] | 0.4605 [5.4] | **0.0018** [5.4] | ***0.0553*** [5.4] | 0.0532 [5.4] |
| Food type (FT) | 0.3259 [2.4] | 0.8633 [3.4] | 0.8587 [2.6] | 0.1165 [2.7] | 0.4937 [2.8] | 0.1818 [3.0] | 0.1100 [2.7] | 0.2877 [2.9] |
| ***Interactions*** |  |  |  |  |  |  |  |  |
| Sex x SW | **0.0128^1^** [4.4] | 0.8788 [5.5] | 0.3476 [5.8] | 0.7947 [4.8] | 0.3939 [5.0] | 0.9917 [5.2] | 0.6107 [4.9] | 0.9788 [5.0] |
| Sex x FT | 0.5557 [2.9] | 0.6283 [4.0] | 0.6276 [3.1] | 0.8636 [3.1] | 0.5920 [3.3] | 0.5473 [3.6] | 0.2567 [3.2] | 0.7704 [3.4] |
| SW x FT | 0.1249 [4.5] | 0.5706 [5.4] | 0.9523 [4.7] | 0.8271 [4.7] | 0.5183 [4.9] | 0.6789 [5.1] | 0.2913 [4.8] | 0.8473 [4.9] |
| Sex x SW x FT | 0.0510 [3.5] | 0.5954 [4.6] | 0.1399 [3.7] | 0.9850 [3.8] | 0.9182 [3.9] | 0.5439 [4.2] | 0.1521 [3.8] | 0.5116 [4.0] |
| % variation explained by participant ID | 67 | 39 | 58 | 57 | 52 | 23 | 55 | 50 |
| **Simplified models** |  |  |  |  |  |  |  |  |
| **1-factor ANOVA** |  |  |  |  |  |  |  |  |
| Sampling week |  |  |  | 0.0037 |  | 0.0010 |  |  |
| **2-factor ANOVA** |  |  |  |  |  |  |  |  |
| Sex | T |  |  |  |  |  |  |  |
| Sampling week (SW) | 0.0019 |  |  |  |  |  |  |  |
| Sex x SW | 0.0204 |  |  |  |  |  |  |  |

| **Legend for Supplementary Table 6.**  VIF, value inflation factors derived from the multicollinearity check (p.lme) in R (a VIF value <5 indicates low correlation, a value between 5 and 10 represents moderate correlation, while a VIF value larger than 10 is a sign for high non-tolerable correlation of model predictors). |
| --- |

| **Supplementary Table 7.** Effects of sex (male, female) and food type (conventional, organic) on plasma concentrations of selected mineral micronutrients (Cu, Fe, Se, Zn) in week 3 of the experiment. Values shown are **main effect means** (95% CI). | | | | |
| --- | --- | --- | --- | --- |
| **Factor** | **Cu**  (µmol/L) | **Fe**  (µmol/L) | **Se**  (µmol/L) | **Zn**  (µmol/L) |
| **Sex** |  |  |  |  |
| Female (n= 26) | **14.3** (12.9,15.8) | **24.7** (22.5,26.9) | **1.19** (1.15,1.23) | **13.0** (12.7,13.3) |
| Male (n= 28) | **11.7** (11.2,12.2) | **27.2** (24.4,30.0) | **1.14** (1.10,1.17) | **12.1** (11.8,12.4) |
| **Food Type** |  |  |  |  |
| Conventional (n=28) | **11.7** (11.2,12.1) | **27.6** (24.4,30.7) | **1.12** (1.09,1.15) | **12.2** (12.0,12.4) |
| Organic (n=26) | **14.4** (13.0,15.8) | **24.3** (22.7,25.9) | **1.21** (1.17,1.24) | **12.9** (12.5,13.2) |
| **ANOVA** *P*-values (VIF) |  |  |  |  |
| ***Main effects*** |  |  |  |  |
| Sex | ***0.0734*** (2.1) | 0.5080 (2.1) | 0.2793 (2.1) | **0.0384** (2.1) |
| Food type (FT) | 0.1543 (2.2) | 0.4697 (2.2) | 0.1401 (2.2) | 0.3224 (2.2) |
| ***Interactions*** |  |  |  |  |
| Sex x FT | 0.4293 (2.6) | 0.4697 (2.6) | 0.8787 (2.6) | 0.9377 (2.6) |
| % variation explained by participant ID | 72 | 72 | 73 | 71 |
| **Simplified models** |  |  |  |  |
| **1-factor ANOVA** |  |  |  |  |
| Main effect Sex |  |  |  | **0.0349** |
| VIF, value inflation factors derived from the multicollinearity check (p.lme) in R (a VIF value <5 indicates low correlation, a value between 5 and 10 represents moderate correlation, while a VIF value larger than 10 is a sign for high non-tolerable correlation of model predictors). | | | | |

| **Supplementary Table 8.** Effects of sex (male, female), sampling week and participant group (intervention, conventional) on the daily urinary excretion of phenolics salicylic acid, toxic metals (Al, Ba, Cd, Pb, Sn) and titanium (Ti). Values shown are **main effect means** (95% CI). | | | | | | | | | | | | | | | |
| --- | --- | --- | --- | --- | --- | --- | --- | --- | --- | --- | --- | --- | --- | --- | --- |
|  | **Phenolic markers**(µmol/day) | |  | **Toxic metals** (nmol/day) | | | | | | | |  | |  | |
| **Factor** | **Total**  **phenolics** | **Salicylic**  **acid** |  | **Al** | **Cd** | **Ni** | | **Pb** | | **Sn** | |  | | **Ti** (nmol/day) | |
| **Sex** |  |  |  |  |  |  | |  | |  | |  | |  | |
| Female (n=104) | **106** (101,111) | **2.8** (2.5,3.0) |  | **321** (281,360) | **2.9** (2.6,3.2) | **80** (74,85) | | **3.8** (3.6,4.1) | | **9.2** (7.0,11.4) | |  | | **1.5** (1.4,1.6) | |
| Male (n=108) | **95** (90,100) | **2.5** (2.3,2.6) |  | **206** (178,233) | **2.3** (2.1,2.4) | **72** (66,77) | | **3.0** (2.7,3.2) | | **4.8** (4.0,5.5) | |  | | **1.2** (1.1,1.3) | |
| **Sampling week** |  |  |  |  |  |  | |  | |  | |  | |  | |
| Wk 1 (n=27) | **76** (70,82) **c** | **2.0** (1.8,2.2) **c** |  | **267** (218,316) | **2.3** (2.0,2.6) | **59** (55,63) **b** | | **2.7** (2.4,2.9) **b** | | **5.3** (3.7,7.0) | |  | | **1.2** (1.0,1.3) | |
| Wk 2 (n=27) | **120** (113,126) **a** | **3.3** (3.0,3.7) **a** |  | **247** (209,285) | **2.7** (2.5,2.8) | **88** (80,96) **a** | | **3.5** (3.2,3.8) **ab** | | **4.7** (3.7,5.7) | |  | | **1.6** (1.4,1.7) | |
| Wk 3 (n=27) | **111** (105,118) **ab** | **2.9** (2.7,3.1) **ab** |  | **285** (238,333) | **2.2** (2.1,2.4) | **84** (77,91) **a** | | **3.7** (3.3,4.0) **a** | | **11.2** (7.4,14.9) | |  | | **1.4** (1.3,1.5) | |
| Wk 5 (n=27) | **94** (87,101) **bc** | **2.2** (1.9,2.4) **bc** |  | **244** (184,305) | **3.0** (2.5,3.6) | **71** (62,80) **ab** | | **3.8** (3.3,4.2) **a** | | **8.1** (5.3,11.0) | |  | | **1.3** (1.1,1.5) | |
| **Participant group** |  |  |  |  |  |  |  | |  | |  | |  | |  |
| Conventional (n=108) | **100** (95,105) | **2.6** (2.4,2.8) |  | **225** (202,248) | **2.2** (2.1,2.3) | **75** (71,80) | **3.5** (3.3,3.7) | | **6.5** (5.0,7.9) | |  | | **1.3** (1.2,1.4) | |  |
| Intervention (n=104) | **100** (95,105) | **2.6** (2.4,2.8) |  | **294** (253,336) | **2.9** (2.6,3.2) | **76** (70,81) | **3.3** (3.0,3.5) | | **7.4** (5.5,9.2) | |  | | **1.4** (1.3,1.6) | |  |
| **ANOVA** *P*-values [VIF] |  |  |  |  |  |  |  | |  | |  | |  | |  |
| ***Main effects*** |  |  |  |  |  |  |  | |  | |  | |  | |  |
| Sex | 0.2509  [4.2; 1.0**^1^**] | 0.4241  [4.4; 1.1**^1^**] |  | 0.0525  [6.0; 1.1**^1^**] | 0.0984  [6.1; 1.1**^1^**] | 0.4224  [4.6; 1.1**^1^**] | ***0.0812***  [4.3; 1.1**^1^**] | | ***0.0794***  [6.4; 1.0**^1^**] | |  | | 0.1187  [5.3; 1.0**^1^**] | |  |
| Sampling Week (SW) | **<0.0001^2^**  [>10; 1.0**^1^**] | **0.0001^2^**  [>10, 1.0**^1^**] |  | 0.8964  [>10; 1.1**^1^**] | 0.1510  [>10; 1.0**^1^**] | **0.0057^2^**  (>10; 1.0**^1^**) | **0.0216^2^**  (>10; 1.0**^1^**) | | 0.3821  (>10; 1.0**^1^**) | |  | | 0.1941  (>10; 1.0**^1^**) | |  |
| Participant group (PG) | 0.7731  [4.2; 1.0**^1^**] | 0.7811  [4.4, 1.1**^1^**] |  | 0.0810  [6.0; 1.1**^1^**] | **0.0284^3^**  [6.1; 1.1**^1^**) | 0.8224  [4.6; 1.1**^1^**] | 0.9486  [4.3; 1.1**^1^**] | | 0.5118  [6.4; 1.0**^1^**] | |  | | 0.1904  [5.3; 1.0**^1^**] | |  |
| ***Interactions*^4^** |  |  |  |  |  |  |  | |  | |  | |  | |  |
| Sex x SW x PG | 0.6429  (>10) | **0.0238^5^**  **(>**10) |  | ***0.0589*** (>10) | 0.2389  (>10) | 0.2851  (>10) | 0.8325  (>10) | | 0.9296  (>10) | |  | | 0.3877  (>10) | |  |
| % variation explained by participant ID | 43 | 40 |  | 30 | 29 | 39 | 42 | | 0.01 | |  | | 34 | |  |
| **Simplified models** |  |  |  |  |  |  |  | |  | |  | |  | |  |
| **1-factor ANOVA** |  |  |  |  |  |  |  | |  | |  | |  | |  |
| Main effect SW | **<0.0001** |  |  |  |  | **0.0039** | **0.0176** | |  | |  | |  | |  |
| Main effect PG |  |  |  |  | 0.1008 |  |  | |  | |  | |  | |  |
| **Legend for Supplementary Table 8.**  VIF, value inflation factors derived from the multicollinearity check (p.lme) in R (a VIF value <5 indicates low correlation, a value between 5 and 10 represents moderate correlation, while a VIF value larger than 10 is a sign for high non-tolerable correlation of model predictors. Means in the same column labelled with the same letter are not significantly different according to Tukey contrasts (general linear hypothesis test; *P <* 0.05). **^1^**, VIP when interaction terms were removed from the model; **^2^**, see Figures 1 and 2 for Holm-adjusted *P*-values; **^3^**, the Holm adjusted *P*-value for participant group is 0.2272; **^4^**, *P*-values for 2-way interactions are not shown, because no significant or trends (0.1>*P*>0.05) towards significant 2-way interactions were detected; **^5^**, see Supplementary Table 10 for interaction means ±SE and the Holm-adjusted *P*-value for the interaction | | | | | | | | | | | | | | |  |

| **Supplementary Table 9.** Effects of sex (male, female), sampling week and participant group (intervention, conventional) on the daily urinary excretion **(**nmol/day unless stated otherwise) of mineral micronutrients (Co, Cr, Cu, I, Mn, Mo, Se, Zn). Values shown are **main effect means** (95% CI). | | | | | | | | |
| --- | --- | --- | --- | --- | --- | --- | --- | --- |
| **Factor** | **Co** | **Cr** | **Cu** | **I** (µmol/d) | **Mn** | **Mo** | **Se** | **Zn** (µmol/d) |
| **Sex** |  |  |  |  |  |  |  |  |
| Female (n=104) | **21** (19,24) | **8.6** (8.0,9.1) | **169** (159,179) | **1.5** (1.3,1.7) | **7.6** (6.5,8.7) | **606** (546,666) | **352** (330,375) | **4.1** (3.8,4.4) |
| Male (n=108) | **13** (11,15) | **8.0** (7.6,8.5) | **142** (136,149) | **1.3** (1.2,1.4) | **5.7** (5.0,6.3) | **617** (567,667) | **342** (322,361) | **5.4** (5.1,5.8) |
| **Sampling week** |  |  |  |  |  |  |  |  |
| Week 1 (n=27) | **9** (8,11) **c** | **7.9** (7.4,8.4) **b** | **172** (157,187) **a** | **1.0** (0.9,1.1) | **5.0** (4.3,5.8) **b** | **674** (580,769) | **320** (289,350) **bc** | **5.2** (4.7,5.8) **a** |
| Week 2 (n=27) | **19** (16,22) **b** | **10.3** (9.6,11.0) **a** | **162** (153,171) **ab** | **1.4** (1.3,1.5) | **9.0** (7.6,10.5) **a** | **754** (686,822) | **387** (368,406) **ab** | **5.6** (5.1,6.0) **a** |
| Week 3 (n=27) | **28** (23,32) **a** | **9.6** (9.0,10.2) **ab** | **160** (150,169) **ab** | **1.7** (1.6,1.8) | **10.1** (8.6,11.6) **a** | **527** (478,576) | **432** (401,462) **a** | **4.7 (**4.2,5.1) **ab** |
| Week 5 (n=27) | **12** (10,14) **bc** | **5.3** (4.8,5.8) **c** | **127** (116,139) **b** | **1.4** (1.0,1.8) | **2.2** (2.0,2.4) **b** | **491** (410,572) | **249** (224,274) **c** | **3.7** (3.2,4.1) **b** |
| **Participant group** |  |  |  |  |  |  |  |  |
| Conventional (n=108) | **16** (14,18) | **8.2** (7.7.8.6) | **157** (148,166) | **1.2** (1.1,1.3) | **6.1** (5.3,6.8) | **663** (597,729) | **357** (336,379) | **4.7** (4.3,5.0) |
| Intervention (n=104) | **18** (15,20) | **8.4** (7.9,8.9) | **154** (146,162) | **1.6** (1.4,1.8) | **7.1** (6.1,8.1) | **564** (523,605) | **337** (317,358) | **4.9** (4.6,5.2) |
| **ANOVA** *P*-values [VIF] |  |  |  |  |  |  |  |  |
| ***Main effects*** |  |  |  |  |  |  |  |  |
| Sex | ***0.0755***  [3.2; 1.1**^1^**] | 0.3989  [7.7; 1.0**^1^**] | 0.1004  [4.8; 1.1**^1^**] | 0.2761  [6.3; 1.1**^1^**] | 0.1603  [6.0; 1.1**^1^**] | 0.8925  [7.9; 1.1**^1^**] | 0.7073  [7.6; 1.1**^1^**] | ***0.0564***  [4.3; 1.1**^1^**] |
| Sampling Week (SW) | **<0.0001^2^**  [>10; 1.0**^1^**] | **<0.0001^2^**  [>10; 1.0**^1^**] | **0.0121^2^**  [>10; 1.0**^1^**] | 0.1119  [>10; 1.0**^1^**] | **<0.0001^2^**  [>10; 1.0**^1^**] | ***0.0554***  [>10; 1.0**^1^**] | **<0.0001^2^**  [>10; 1.0**^1^**] | **0.0036^2^**  [>10; 1.0**^1^**] |
| Participant Group (SG) | 0.4401  [3.2; 1.0**^1^**] | 0.5669  [7.7; 1.0**^1^**] | 0.8254  [4.8; 1.1**^1^**] | ***0.0731***  [6.3; 1.1**^1^**] | 0.2527  [6.0; 1.1**^1^**] | 0.2108  [7.9; 1.1**^1^**] | 0.5191  [7.6; 1.1**^1^**] | 0.8711  [4.3; 1.1**^1^**] |
| ***Interactions*** |  |  |  |  |  |  |  |  |
| Sex x SW | **0.0169^3,5^** | 0.9902**^3^** | 0.4166**^3^** | 0.1854**^3^** | ***0.0929*^3^** | 0.8543**^3^** | 0.6142**^3^** | 0.9786**^3^** |
| Sex x PG | 0.6096**^4^** | 0.1691**^3^** | 0.4164**^4^** | 0.4734**^3^** | 0.3581**^3^** | 0.7816**^3^** | **0.0122^3,6^** | 0.3709**^4^** |
| SW x PG | ***0.0931*^3^** | 0.5533**^3^** | 0.9866**^3^** | 0.2584**^3^** | 0.7565**^3^** | 0.9550**^3^** | 0.1104**^3^** | 0.2764**^3^** |
| Sex x SW x PG | 0.2747**^3^** | 0.7752**^3^** | 0.3277**^3^** | 0.2483**^3^** | 0.8187**^3^** | 0.4309**^3^** | 0.6273**^3^** | 0.7001**^3^** |
| % variation explained by participant ID | 54 | 18 | 38 | 28 | 30 | 16 | 19 | 42 |
| **Simplified model** |  |  |  |  |  |  |  |  |
| **1-factor ANOVA** |  |  |  |  |  |  |  |  |
| Sampling week |  | **<0.0001** | **0.0100** |  | **<0.0001** |  |  | **0.0026** |

| **Legend for Supplementary Table 9.**  VIF, value inflation factors derived from the multicollinearity check in R (a VIF value <5 indicates low correlation, a value between 5 and 10 represents moderate correlation, while a VIF value larger than 10 is a sign for high non-tolerable correlation of model predictors. Means in the same column labelled with the same letter are not significantly different according to Tukey contrasts (general linear hypothesis test; *P <* 0.05).  **^1^**, VIP when interaction terms were removed from the model; **^2^**, see Figures 1 and 2 for Holm-adjusted *P*-values; **^3^** VIF is high (>10); **^4^**, VIF is moderate (between 5 and 10); **^5^**.see Supplementary Table 11 for interaction means ±SE and the Holm-adjusted *P*-value for the interaction; **^6^** see Supplementary Table 12 for interaction means ±SE and the Holm-adjusted *P*-value for the interaction |
| --- |

| **Supplementary Table 10.** Effect of sex, sampling week and study participant urinary excretion of salicylic acid (µmol/day). Values shown are **interaction^1^ means** ±SE. | | | | |
| --- | --- | --- | --- | --- |
|  |  |  | **Factor 3. Participant group** | |
| **Factor 1. Sampling week** | **Factor 2. Sex** |  | **Conventional** | **Intervention** |
| **Wk1** | Female |  | **1.7** ±0.3 **A a**  (n=5) | **1.8** ±0.4 **A a**  (n=8) |
|  | Male |  | **1.4** ±0.4 **A a**  (n=9) | **2.7** ±0.5 **A a**  (n=5) |
|  |  |  |  |  |
| **Wk2** | Female |  | **4.1** ±0.8 **A a**  (n=5) | **2.7** ±0.9 **A a**  (n=8) |
|  | Male |  | **2.0** ±0.6 **B b**  (n=9) | **3.7** ±0.4 **A a**  (n=5) |
|  |  |  |  |  |
| **Wk3** | Female |  | **3.5** ±0.5 **A a**  (n=5) | **2.7** ±0.5 **A a**  (n=8) |
|  | Male |  | **2.5** ±0.5 **A a**  (n=9) | **2.7** ±0.5 **A a**  (n=5) |
|  |  |  |  |  |
| **Wk4** | Female |  | **2.4** ±0.5 **A a**  (n=5) | **2.7** ±0.5 **A a**  (n=8) |
|  | Male |  | **2.2** ±0.9 **A a**  (n=9) | **1.6** ±0.2 **A a**  (n=5) |
| **^1^**, the *P*-value for the 3-way interaction is 0.0238 and the Holm-adjusted *P*-value is 0.1906.  In the same sampling week, interaction means for the two sexes followed by the same lower-case letter and the two study groups followed by the same capital letters are not significantly different, according to Tukey contrasts (general linear hypothesis test; *P<*0.05). | | | | |

| **Supplementary Table 11.** Effect of sex and sampling week on urinary excretion of cobalt (Co) (nmol/day). Values shown are **interaction^1^ means** ±SE | | | | | |
| --- | --- | --- | --- | --- | --- |
|  |  | **Factor 2. Sampling week** | | | |
| **Factor 1. Sex** |  | **Wk1** | **Wk2** | **Wk3** | **Wk4** |
| Female |  | **11** ±3 **C a**  (n=13) | **23** ±4 **B a**  (n=13) | **37** ±6 **A a**  (n=13) | **15** ±3 **C a**  (n=13) |
| Male |  | **8** ±3 **B a**  (n=14) | **16** ±5 **A a**  (n=14) | **18** ±5 **A b**  (n=14) | **9** ±3 **B a**  (n=14) |
| The *P*-value for the interaction is 0.0169 and the Holm adjusted *P*-value is 0.1354.  Interaction means for different sexes in the same sampling week followed by the same lower-case letter and different sampling weeks for the same sex followed by the same capital letters are not significantly different, according to Tukey contrasts (general linear hypothesis test; *P<*0.05). | | | | | |

| **Supplementary Table 12.** Effect of sex and participant group on urinary excretion of selenium (Se) (nmol/day). Values shown are **interaction^1^ means** ±SE | | | |
| --- | --- | --- | --- |
|  |  | **Factor 2. participant group** | |
| **Factor 1. Sex** |  | **Conventional** | **Intervention** |
| Female |  | **329** ±24 **ab**  (n=20) | **390** ±44 **a**  (n=32) |
| Male |  | **403** ±39 **a**  (n=36) | **307** ±20 **b**  (n=20) |
| The P-value for the interaction is 0.0122 and the Holm adjusted P-value is 0.0978.  Interaction means followed by the same lower-case letter are not significantly different, according to Tukey contrasts (general linear hypothesis test; *P<*0.05). | | | |

| **Supplementary Table 13.** Effects of sex (male, female), sampling week and participant group (intervention, conventional) on plasma concentrations of selected mineral micronutrients (Cu, Fe, Se, Zn) in weeks 1, 3 and 5 of the experiment. Values shown are **main effect means** (95% CI). | | | | |
| --- | --- | --- | --- | --- |
| **Factor** | **Cu**  (µmol/L) | **Fe**  (µmol/L) | **Se**  (µmol/L) | **Zn**  (µmol/L) |
| **Sex** |  |  |  |  |
| Female (n= 26) | **14.4** (13.7,15.1) | **25.4** (23.7,27.0) | **1,14** (1.12,1.17) | **13.1** (12.9,13.4) |
| Male (n= 28) | **11.8** (11.5,12.03) | **25.0** (23.8,26.2) | **1.08** (1.06,1.10) | **12.3** (12.2,12.5) |
| **Sampling week** |  |  |  |  |
| Wk 1 (n=27) | **12.7** (12.1,13.4) | **24.4** (22.5,26.4) | **1.02** (0.99,1.1) **b** | **12.6** (12.3,12.9) |
| Wk 3 (n=27) | **13.0** (12.3,13.6) | **26.0** (24.0,27.9) | **1.16** (1.13,1.19) **a** | **12.5** (12.2,12.8) |
| Wk 5 (n=27) | **13.5** (12.8,14.1) | **25.2** (23.6,26.7) | **1.15** (1.1,1.2) **a** | **13.0** (12.7,13.2) |
| **Participant group** |  |  |  |  |
| Conventional (n=28) | **14.2** (13.5 - 14.92) | **25.8** (24.3,27.4) | **1.15** (1.13,1.17) | **12.9** (12.7,13.2) |
| Intervention (n=26) | **12.0** (11.7 - 12.24) | **24.6** (23.2,26.0) | **1.08** (1.06;1.10) | **12.5** (12.4,12.7) |
| **ANOVA** *P*-values (VIF) |  |  |  |  |
| ***Main effects*** |  |  |  |  |
| Sex | **0.0376** (2.4, 1.1**^1^**) | 0.8842 (2.4; 1.1**^1^**) | 0.1745 (2.8; 1.1**^1^**) | **0.0414** (4.0; 1.1**^1^**) |
| Sampling Week (SW) | ***0.0708*** (>10; 1.0**^1^**) | 0.8201 (>10; 1.0**^1^**) | **<0.0001** (>10; 1.0**^1^**) | 0.2644 (>10; 1.0**^1^**) |
| Participant group (PG) | 0.1963 (2.4; 1.1**^1^**) | 0.6014 (2.4; 1.1**^1^**) | 0.1896 (2.8; 1.1**^1^**) | 0.6360 (4.0; 1.1**^1^**) |
| ***Interactions*** |  |  |  |  |
| Sex x SW | 0.7339 (>10) | 0.3934 (>10) | 0.7264 (>10) | 0.3080 (>10) |
| Sex x PG | 0.4391 (4.2) | 0.7441 (3.1) | 0.6893 (4.9) | 0.1552 (7.0) |
| SW x PG | 0.4233 (>10) | 0.3704 (>10) | 0.5993 (>10) | 0.4364 (>10) |
| Sex x SW x PG | 0.5022 (>10) | 0.8765 (>10) | 0.5874 (>10) | 0.2274 (>10) |
| % variation explained by participant ID | 72 | 25 | 59 | 39 |
| **Simplified models** |  |  |  |  |
| **1-factor ANOVA** |  |  |  |  |
| Main effect Sex | **0.0381** |  |  | **0.0419** |
| Main effect SW |  |  | **0.0010** |  |
| VIF, value inflation factors derived from the multicollinearity check (p.lme) in R (a VIF value <5 indicates low correlation, a value between 5 and 10 represents moderate correlation, while a VIF value larger than 10 is a sign for high non-tolerable correlation of model predictors. **^1^**, VIP when interaction terms were removed from the model; | | | | |

| **Enrolment** |  | | **Assessed for eligibility* (n =30)** | | | | | | | | | |  | |  |
| --- | --- | --- | --- | --- | --- | --- | --- | --- | --- | --- | --- | --- | --- | --- | --- |
|  |  | |  | | | |  |  |  | | | |  | |  |
|  |  | |  | | | |  |  | **Excluded (n=3)** | | | | | | |
|  |  | |  | | | |  | **🡪** | - Declined to participate (n=3) | | | | | | |
|  |  | |  | | | |  |  |  | | | |  | |  |
|  |  | |  | | **Randomized (n=27)** | | | | | |  | |  | |  |
|  |  | |  | | | |  | **🡫** |  | | | |  | |  |
|  |  |  |  | | | |  | |  | | | |  |  |  |
|  | **🡫** |  |  |  | | **Allocation** | | | |  | |  |  | **🡫** |  |
| **Allocated to conventional group (n = 14)** | | | | | | |  | | **Allocated to intervention group (n = 13)** | | | | | | |
|  |  |  |  | | | |  | |  | | | |  |  |  |
|  | **🡫** |  |  |  | | **Analysis** | | | |  | |  |  | **🡫** |  |
| **Analyzed (n = 14)** | | | | | | |  | | **Analyzed (n = 13)** | | | | | | |
|  |  | |  | | | |  | |  | | | |  | |  |
|  |  | |  | | | |  | |  | | | |  | |  |
| **Supplementary Figure 1.** Flow diagram of the study design based on CONSORT 2010 guidelines.  * all 30 postgraduate students that registered for the Newcastle University field course on Perennial crop production in 2017 were eligible. | | | | | | | | | | | | | | | |

| 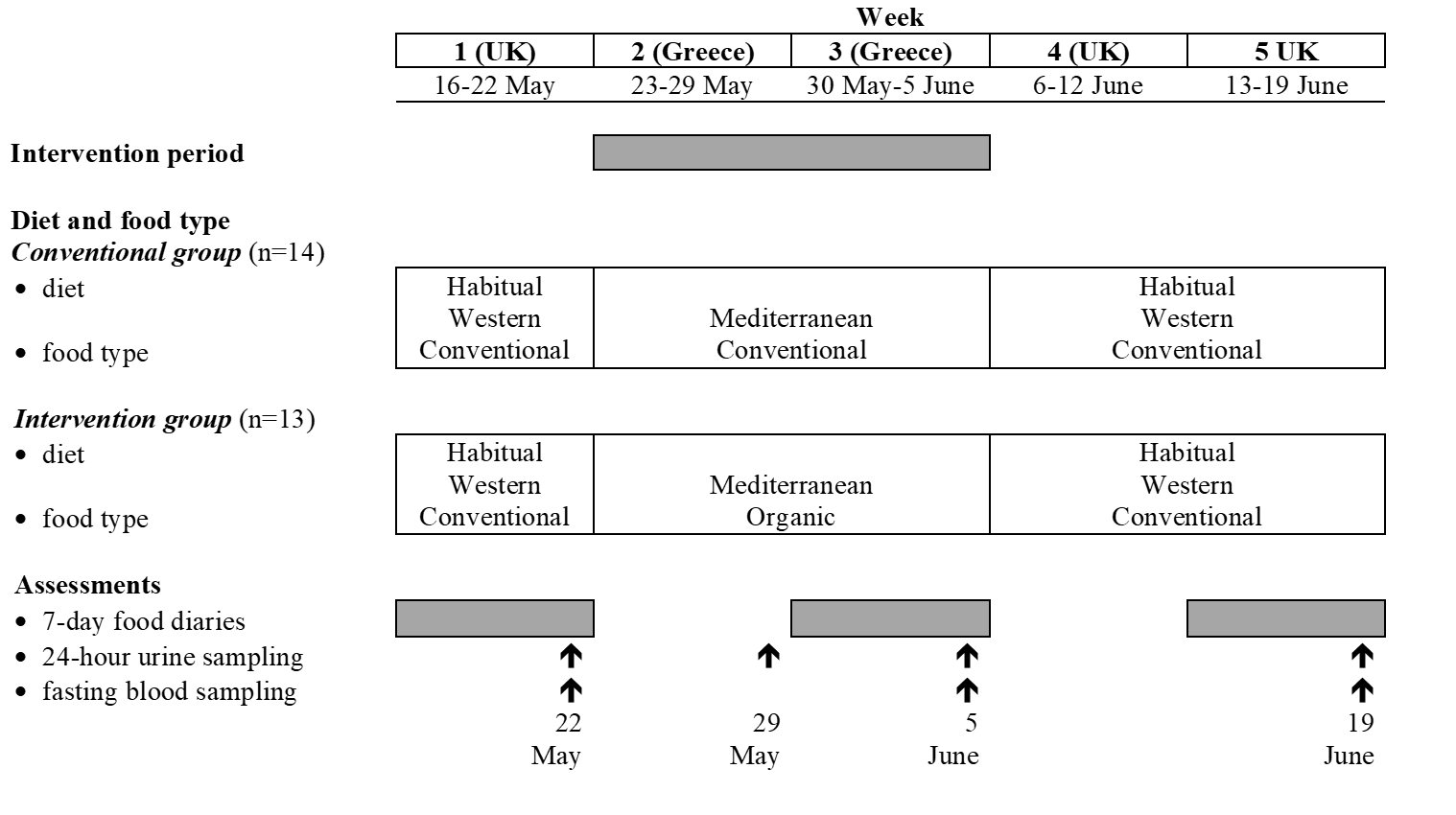 |
| --- |
| **Supplementary Figure 2.** Study design; diet and food types (organic vs conventional) consumed and assessments carried out before, during and after the intervention period. |
